# Data-Driven Virtual Reality Environment Recommendation for Long-Term Care Residents With Major Neurocognitive Conditions: Feasibility and Proof-of-Concept Study

**DOI:** 10.2196/101192

**Published:** 2026-11-12

**Authors:** Alejandro Reyes-Consuelo, Jocelyne Kiss, Julien Voisin

**Affiliations:** 1 School of Design, Faculty of Planning, Architecture, Art and Design, Laval University, 2325, rue de l'Université, Québec, QC, G1V 0A6, Canada; 2 Center of Interdisciplinary Research in Rehabilitation and Social Integration (CIRRIS), Québec, QC, Canada; 3 International Observatory on the Societal Impacts of AI and Digital Technologies (OBVIA), Québec, QC, Canada; 4 School of Rehabilitation Sciences, Faculty of Medicine, Laval University, Québec, QC, Canada

**Keywords:** dementia, virtual reality, clinical decision support, heart rate variability, machine learning, long-term care, nonpharmacological intervention

## Abstract

**Background:**

Virtual reality (VR) comfort sessions are increasingly used in residential dementia care, but environment selection remains ad hoc—staff have no systematic way to identify which virtual experience best suits a given resident.

**Objective:**

This feasibility and proof-of-concept study aimed to determine whether a pipeline integrating wearable physiological signals (heart rate variability and electrodermal activity), behavioral observations, and semantic environment characteristics can detect a person-specific, environment-level signal usable for between-session VR environment recommendation in long-term care and whether the resulting recommendations can be communicated to care staff and families through plain-language reports.

**Methods:**

Twenty-one residents with major neurocognitive conditions received 420 immersive VR sessions (5 environments, 2 sessions per week, 10 weeks) in a long-term care facility. We defined an operational hybrid calm target requiring behavioral calm (Pittsburgh Agitation Scale vocal intensity score of 0) and root mean square of successive differences at or above the participant’s personal median to address the ceiling effect of behavioral measures alone (sessions already rated calm: 234/358, 65%). A compact 23-feature model (10 physiological, 3 behavioral, and 10 environment) was selected from more than 2100 configurations on the basis of cross-validation error and interpretability; the environment-level signal was tested with an environment permutation test on held-out sessions (weeks 8‐10) and in sensitivity analyses.

**Results:**

Environment features carried a statistically significant but borderline signal for predicting hybrid calm (environment permutation test, *P*=.045 with 200 permutations; *P*=.04 with 2000 permutations; Cohen *d*=1.75); 4 alternative models using 344 to 368 features ignored environment entirely (all *P*>.79). Person-level effects dominated prediction variance (partial η²=0.42; *P*<.001); environment-level discrimination was modest (pairwise ranking accuracy 57.9% vs 50% chance; top-1 hit rate 27.8%, 95% CI 19.8%‐35.7%, including chance). Water presence was the most influential environment characteristic. In sensitivity analyses, the calm label was robust to the threshold (κ=0.79‐0.89), but the environment permutation significance was not: under alternative thresholds and a continuous target, the effect kept its direction (*d*=0.65‐1.60) without reaching significance (*P*=.06‐.57), and a selection criterion computed on training period data only did not select the compact model. The pipeline generated 60 evidence-traced plain-language reports (30 family and 30 staff); all passed automated safety checks, with staff reports showing higher data fidelity and family reports showing better nonmedical framing.

**Conclusions:**

An end-to-end pipeline from wearable sensor to plain-language report is technically feasible in residential dementia care. The environment-level signal is suggestive—consistent in direction across target definitions and seeds—but its significance was specific to one labeling of one configuration whose selection was partly informed by the test set; it is exploratory evidence requiring independent replication, not a demonstration that the recommendations improve resident outcomes. Prospective validation against resident outcomes is required before any use in care decisions.

## Introduction

### Background

Virtual reality (VR) is increasingly used in residential dementia care to offer structured, immersive experiences (eg, nature scenes, familiar places, and guided activities) within short sessions that fit the operational constraints of long-term care settings [1-3]. The rationale is not clinical treatment in the conventional sense but day-to-day support: facilitating engagement, offering moments of calm or interest, and providing structured activities during periods when behavioral and psychological symptoms of dementia (BPSD) might otherwise escalate [4]. A growing body of evidence suggests that such sessions can reduce agitation-related behaviors, improve momentary affect, and are generally well tolerated, although findings remain heterogeneous across protocols, populations, and measurement approaches [5,6].

A persistent challenge, however, is that responses to VR are heterogeneous both between and within individuals. A resident may appear comfortable during one session and restless during another, even when the content seems similar. The same virtual environment can be experienced differently depending on fatigue, momentary stressors, sensory sensitivity, or cognitive fluctuation [7]. This variability complicates routine practice: care staff typically rely on memory, informal notes, or rotating schedules to select which environment to present, with no systematic mechanism for detecting or acting on individual preferences. The broader nonpharmacological intervention literature has repeatedly identified personalization—tailoring activities to the individual’s history, preferences, and current state—as a key determinant of effectiveness [8,9].

Personalized adaptive interventions are an established class in behavioral health: just-in-time adaptive interventions adjust the type, timing, or amount of support to an individual’s changing internal and contextual state, drawing on continuous data streams to tailor delivery [10]. Within dementia care specifically, algorithmic individualization has empirical support—Cohen-Mansfield et al [11] found that nonpharmacological interventions matched to each resident’s unmet needs reduced agitation in nursing home residents in a placebo-controlled trial, and the Tailored Activity Program (250-dyad randomized controlled trial [RCT]) showed that occupational therapist–led activity tailoring improved functional outcomes and caregiver well-being [12]. These precedents rely on human-administered tailoring at the authoring or delivery stage. This work contributes a data-driven environment recommendation mechanism in that lineage, supporting between-session decisions by care staff rather than executing within-session content adaptation.

Within VR specifically, Li et al [13] provide a recent systematic review of 132 studies on personalized interaction in VR. They describe a 5-stage closed-loop framework for real-time adaptive systems (input collection, data processing, adaptive logic, system update, and feedback loop) and distinguish real-time adaptation, in which the system modifies the experience dynamically during a session, from offline adaptation, in which models use previously collected data to inform subsequent interactions. They further categorize adaptive objectives into 4 types: difficulty adjustment, display adaptation, environment manipulation, and feedback personalization. Across the 132 reviewed studies, most systems operate in real time, rely on machine learning adaptation of physiological and behavioral signals (notably electroencephalography [EEG], galvanic skin response [GSR] or electrodermal activity [EDA], heart rate [HR] or heart rate variability [HRV], and ocular data), and concentrate in mental health, education and training, gaming, and rehabilitation domains, targeting cognitively intact users. Residential long-term care for residents with major neurocognitive conditions is not a represented application domain in their review, and between-session environment selection from a curated library—as distinct from within-session environment manipulation—is not an explicit category within their taxonomy.

Within dementia care specifically, personalization has developed along 2 largely separate tracks. The first track creates memory-specific custom content through qualitative elicitation with residents and families, either by filming personally meaningful locations [14,15] or curating scene libraries from family-supplied materials [16]; this personalizes content at the authoring stage but does not use a resident’s own session-to-session response data to choose between preauthored options. The second applies person-specific machine learning to wearable physiological and behavioral data for BPSD detection—Iaboni et al [7] showed that person-specific multimodal models classify agitation episodes substantially better than population-level models, with the most predictive features varying across residents. These detection models answer “Is this resident agitated now?” rather than “Which environment is most likely to keep them calm next session?”—they do not select interventions. HRV in particular has been independently validated as an indicator of autonomic dysregulation and agitation propensity in Alzheimer disease [17,18]. The gap is specific: no system combines person-specific physiological modeling with semantic environment characteristics for data-driven between-session environment recommendation from a curated VR library in residents with major neurocognitive conditions.

The absence of systematic environment selection carries a direct clinical cost. In long-term care, BPSD, including agitation, vocal distress, and social withdrawal, are among the primary drivers of pharmacological intervention, despite guidelines that prioritize nonpharmacological approaches as first-line responses. A poorly matched activity is not merely neutral: it can actively increase distress or accelerate behavioral escalation. In the context of VR, assigning environments by rotating schedule or staff memory risks delivering sessions that produce no benefit or cause discomfort, with no mechanism to detect this mismatch or correct it over time. Two constraints specific to this population sharpen the problem. First, residents with major neurocognitive conditions cannot reliably self-report preferences. Second, behavioral observation alone is insufficient to distinguish comfort from passive withdrawal, a ceiling effect that limits what staff can infer from watching a session. The data needed to improve selection (physiological signals from wearable sensors, behavioral ratings from trained observers, and the semantic characteristics of the VR content itself) are either already collected or collectable at low additional cost during routine sessions.

Physiological wearables offer a complementary, continuous trace of the body’s response during a session. Photoplethysmography (PPG)–derived HRV and EDA can provide time-resolved indicators of autonomic state and arousal regulation without requiring the resident to articulate a subjective experience. HRV, in particular, has been linked to emotion regulation capacity and agitation propensity in Alzheimer disease [17,18], and wearable multimodal sensors have shown promise for detecting behavioral symptoms in dementia care units [7]. However, these signals are notoriously difficult to use in real-world care environments: devices are worn inconsistently, motion artifacts are frequent, recordings contain gaps, and exports are often incomplete [19]. Moreover, observer-rated behavioral measures such as the Pittsburgh Agitation Scale (PAS) remain subjective and coarse; in our own data, nearly two-thirds of sessions were already rated as behaviorally calm, leaving limited room for between-environment differentiation using behavioral scores alone. Combining physiological and behavioral information into a stronger, convergent indicator of comfort is both a methodological opportunity and a necessity.

At the same time, even a well-performing predictive model is of limited clinical value if its outputs cannot be communicated to the people who make care decisions. Families want to know how their relative experienced a session; staff need structured summaries that support documentation and planning. Large language models (LLMs) have demonstrated the ability to generate fluent, structured text from numerical inputs, but their application to dementia care reporting, where nonmedical framing, evidence tracing, and avoidance of clinical overclaiming are essential, has not been explored.

### VR in Residential Dementia Care

Immersive VR is being explored in long-term care as a way to deliver structured sensory experiences (nature exposure, cultural scenes, reminiscence cues, and light interactive activities) to residents who can no longer access such experiences in the physical world [6,20]. Sessions are typically brief (5‐20 min), supervised by staff, and delivered through head-mounted displays. The therapeutic rationale is not cognitive rehabilitation but moment-to-moment well-being: facilitating engagement, eliciting positive affect, and reducing the frequency or severity of BPSD during and after exposure [5,21].

Evidence on outcomes remains mixed. A recent systematic review and meta-analysis found that VR may reduce depressive symptoms and aggression but reported no significant effect on agitation as measured by standard instruments, with high heterogeneity across included studies [5]. An RCT in acute care found that VR significantly reduced aggressive behavior and loud vocalizations but no other BPSD domains [1]. Qualitative studies consistently report that residents enjoy the experiences and that sessions can prompt social interaction with staff and family members [22,23]. Feasibility is generally supported: headsets are tolerated by most residents, including those with moderate-to-severe dementia, although comfort, fit, and cybersickness remain concerns for subsets of users [20,24].

A construct central to VR research but rarely examined in dementia care is the sense of presence—the subjective experience of being in the virtual environment rather than in the room where the headset is worn. Presence is conventionally assessed with postexposure questionnaires, which residents with major neurocognitive conditions cannot reliably complete. Neurophysiological approaches that characterize presence from combined electroencephalographic and autonomic recordings [25] and that compare neural and autonomic responses to virtual and physical versions of the same stimulus [26] offer a possible route to objective assessment, although they have so far been validated only in cognitively intact adults. This study did not measure presence; the implications of this omission for interpreting between-environment differences in physiological response are discussed in the Limitations section.

A notable gap in this literature is the absence of systematic personalization. Studies typically expose all participants to the same VR content or allow free selection without structured tracking. Residents who expressed preferences for tailored, culturally relevant content showed greater engagement [22], and intervention research in dementia care more broadly has identified personalization as a key determinant of effectiveness across nonpharmacological approaches [8,9]. Yet no study has used physiological or behavioral data to match specific environments to specific residents. This work addresses this gap directly.

### Physiological Monitoring in Dementia Care Settings

HRV, the beat-to-beat variation in cardiac intervals, is widely used as an index of autonomic nervous system function and, by extension, of emotion regulation capacity [27]. In populations with dementia, reduced HRV has been associated with disease severity, with a systematic review and meta-analysis confirming lower parasympathetic activity in people with dementia compared to healthy controls, particularly for time-domain measures such as root mean square of successive differences (RMSSD) and SD of normal-to-normal RR intervals (SDNN) [17]. More directly relevant to this study, Liu et al [18] used data from the Atherosclerosis Risk in Communities cohort to demonstrate that declining HRV across 2 decades was associated with increased risk of agitation in people with clinically diagnosed Alzheimer disease, supporting the plausibility of HRV as a physiological marker of agitation propensity.

Wearable multimodal sensors, combining PPG for cardiac signals with EDA for sympathetic arousal, have been applied in dementia care units to detect agitation episodes. Iaboni et al [7] fitted 17 residents with wristbands for up to 8 weeks and developed person-specific machine learning models that classified behavioral symptoms with a median area under the curve of 0.87, with feature importance varying substantially across individuals. This work demonstrated the value of person-specific modeling and of combining multiple physiological modalities, but it focused on detecting agitation events rather than predicting comfort or recommending activities.

Beyond dementia care, neurophysiological measurement is increasingly used to evaluate immersive experiences objectively rather than through self-report alone. Recent work has proposed EEG-derived indices to track skill acquisition during virtual training [28], evaluated haptic exoskeletons intended to add force feedback to VR training [29], and combined electroencephalographic and autonomic recordings to compare responses to a virtual and a physical cultural artifact [26], in addition to the work cited earlier [25]. These studies concern cognitively intact adults in training or cultural contexts, but they support the premise of this work, that physiological signals recorded during immersion carry interpretable information about how a user is responding to a virtual environment, while also highlighting how little of this literature has reached residential dementia care.

The practical challenges of physiological monitoring in this population are considerable. Motion artifacts from involuntary movements are common, sensor wear is inconsistent, and recording quality varies across sessions. EDA is particularly prone to gaps and artifacts in older adults with thin, dry skin [7,19]. Any system that claims to use physiological data for clinical decision support must therefore include explicit quality control and must distinguish between measured and reconstructed signals, a principle that guides the preprocessing pipeline described in the Methods section.

A final limitation of existing approaches is reliance on behavioral agitation measures alone as outcomes. Instruments such as the PAS capture observable distress but miss internal states: a resident may appear behaviorally calm while showing elevated sympathetic arousal (withdrawn rather than comfortable) or may vocalize without physiological distress markers (engaged rather than agitated). This ambiguity motivates the hybrid calm target used in this study, which requires convergence of behavioral and physiological indicators.

### Decision Support and Clinical Communication in Dementia Care

Translating predictive model outputs into actionable guidance for care practitioners remains a largely unsolved problem in applied health technology. In dementia care, this challenge is compounded by the need to communicate with 2 distinct audiences: clinical staff, who need structured summaries to support documentation and session planning; and family members, who want accessible, nonmedical accounts of their relative’s experiences [30].

LLMs have demonstrated capabilities in health care report generation, including transforming pathology reports into patient-friendly language [31], drafting responses to patient portal messages [32], and generating clinical summaries from structured data [33]. Evidence suggests that LLM-generated reports can improve patient comprehension and reduce the time clinicians spend on communication tasks [31]. However, concerns about factual accuracy, hallucination, and the risk of medical overclaiming remain central to responsible deployment [34,35]. Large-scale review evidence indicates that despite rapid growth in clinical LLM evaluation studies, rigorous patient-centered evaluations remain scarce and existing evidence is heavily weighted toward simulated or examination-style tasks, justifying continued caution about clinical deployment [36].

In dementia care specifically, no prior work has used language models to generate reports from physiological monitoring or predictive model outputs. This study introduces an evidence-tracing framework in which every factual claim in a generated report is linked to a specific data source (a physiological measurement, a behavioral observation, or a model prediction). This design explicitly constrains the language model to nonmedical framing: the system describes patterns and preferences, never diagnoses, prescribes, or makes clinical recommendations. The evaluation strategy assesses both the accuracy of this evidence tracing and the model’s adherence to nonmedical language boundaries, separately for family-audience and staff-audience reports.

### Operational Definition, Research Questions, and Hypotheses

#### Overview

For this study, we define data-driven VR environment recommendation operationally as the identification, for a given resident, of which environment from a curated finite library is most likely to produce physiological and behavioral calm, based on that resident’s prior response history. The recommendation pipeline (1) uses passively collected physiological signals (HRV and EDA) and behavioral ratings as inputs, (2) chooses between environments rather than modifying content within one, (3) operates at the between-session, offline timescale in the terminology of Li et al [13], and (4) surfaces ranked recommendations to care staff through plain-language reports rather than executing environment changes autonomously. This operational niche is distinct from real-time physiological-adaptive VR, from population-level content recommendation, and from the 2 dementia-specific tracks above (qualitative content authoring and person-specific BPSD detection).

Within this operational definition, a further distinction is important. We differentiate person-level discrimination, the ability to predict a given resident’s overall response tendency (eg, that resident P03 is consistently calm and resident P17 consistently restless), from environment-level discrimination, the ability to predict which specific VR environment, from a finite curated library, is most likely to produce calm for that resident (eg, that resident P10 is calmer in an urban heritage scene than in a forest scene). Person-level discrimination can be achieved by any model that captures between-resident variance in baseline physiological or behavioral state; it does not require environment features. Environment-level discrimination, in contrast, requires the model to use environment characteristics to differentiate between-environment responses within a resident. This study tests whether environment-level discrimination is achievable with the available data, using an ablation comparison between a model that includes environment features and models that do not (see research question [RQ] 1 below). These considerations converge on a single overarching RQ: Can passively collected physiological and behavioral data, combined with semantic characteristics of virtual environments, support data-driven VR environment recommendation for residents with major neurocognitive conditions in long-term care, and can the resulting recommendations be communicated transparently to the people who make care decisions?

This paper presents a decision-support pipeline for data-driven VR environment recommendation in residential dementia care. The pipeline integrates wearable physiological signals recorded during sessions, semantic characteristics of the VR environments, and behavioral observation data to predict a hybrid calm target that requires convergence of behavioral calm and elevated parasympathetic tone. It then ranks environments per resident, validates the environment-level signal through permutation testing, and generates plain-language reports for families and clinical staff using an LLM with evidence tracing.

We evaluated this system in a cohort of 21 residents with major neurocognitive conditions who received 420 immersive VR comfort sessions across 10 weeks in a long-term care facility. From the overarching question, 3 specific subquestions structured the investigation; the first 2 are paired with prospective hypotheses grounded in the reviewed literature, and the third is framed as an exploratory question for reasons detailed below.

#### RQ1 (Environment-Level Discrimination) and Hypothesis 1 (the Environment-Level Signal)

RQ1 is as follows: Can a data-driven model identify which of several VR environments produces the calmest experience for a given resident, using their physiological response profile, environment characteristics, and behavioral observation history?

Two findings motivate hypothesis 1. Iaboni et al [7] established that person-specific physiological signatures exist in this population and are recoverable from wearable data. Li et al [13] cataloged VR systems in which physiological signals drive content adaptation in cognitively intact users. We expected both results to carry over to between-session environment selection in residents with major neurocognitive conditions, but only when the model is given environment characteristics because physiological features alone describe the resident, not the environment.

A predictive model combining physiological signals, behavioral observations, and semantic environment characteristics will produce environment-specific recommendations that differ meaningfully across residents, defined operationally as a statistically significant increase in prediction error when environment features are permuted (*P*<.05), whereas a model using physiological signals alone will produce undifferentiated recommendations that reflect who is calm rather than which environment calms them.

#### RQ2 (Feature Contribution) and Hypothesis 2 (the Hybrid Target)

RQ2 is as follows: Which families of input features, and, specifically, which environment characteristics, contribute most to environment recommendation?

Three considerations motivate hypothesis 2: (1) RMSSD indices parasympathetic activity and emotion regulation capacity [27]; (2) reduced parasympathetic tone has been documented in dementia [17]; and (3) longitudinal HRV decline has been associated with increased agitation risk in Alzheimer disease [18]. Behavioral agitation instruments are subject to ceiling effects in stable care routines (as discussed earlier), empirically confirmed in our cohort, where 65.4% (234/358) of rated sessions scored as behaviorally calm. A convergent behavioral-physiological target should recover between-environment variance that either signal alone would miss.

A hybrid calm target requiring convergence of behavioral calm (a Pittsburgh Agitation Scale, second version [PAS-2], vocal intensity score of 0) and elevated parasympathetic tone (RMSSD at or above the participant’s personal median) will better differentiate between-environment effects than a behavioral target alone, because behavioral observation is subject to a ceiling effect that masks physiological variability in this population.

#### Hypothesis 3 (Water Presence as the Primary Environment Differentiator)

Four considerations motivate hypothesis 3. Aquatic natural scenes produce measurable physiological signatures of relaxation [37,38], with systematic review evidence supporting positive associations between blue-space exposure and mental health [39]. The effect appears particularly pronounced in older adults [38]. Tanja-Dijkstra et al [40] showed that it transfers to immersive media: VR beach walks produced greater anxiety reductions than VR city walks in a randomized trial. Aquarium exposure has likewise been reported to have calming effects in dementia care [38]. Among our 5 environments, water presence is the semantic dimension with the greatest between-environment contrast (Table 1: forest and stream, 1.0; beach, 0.67; and Old Québec, 0), so if blue-space effects transfer at all to our set, this is the feature on which between-environment differences should load most strongly, although we expected the effect to be person specific rather than uniform.

**Table 1.** Semantic environment encoding (ordinal ratings normalized to [0, 1]).

Feature	Beach	Île d’Orléans	Forest and stream	Old Québec	Active forest
Motion					
Camera	0.33	1	1	0.67	0.67
Scene	0.33	0.33	0.67	0.67	0.67
Audio					
Nature	0.33	0	0.67	0.33	0.67
Music	0	1	0	0	0
Social presence	0.33	0	0.33	1	0
Visual openness	0.67	0.67	0.33	0.33	0.33
Water presence	0.67	0.33	1	0	0.33
Interaction demand	0	0	0	0	0.33
Visual complexity	0.33	0.67	1	0.67	1
Altitude	0.33	0.67	0.33	0.33	0.33

Among the semantic environment characteristics, water presence will be the strongest predictor of between-environment differences in the hybrid calm target, reflecting the established association between blue-space exposure and indicators of autonomic relaxation, including in older adult populations [38,39], an effect expected to be person-specific rather than uniform across all residents.

RQ3 (Clinical Communication)

RQ3 is as follows: Can an LLM generate evidence-traced, nonmedical reports that communicate model recommendations to families and clinical staff?

RQ3 is treated as exploratory. The relevant prior literature on LLM-generated clinical summaries, physician-patient communication, and safety-constrained text generation in health care does not extend to the specific case of dementia care reporting on VR comfort sessions and offers no directional prediction about quantities of primary interest here, including the expected trade-off between numerical fidelity (how faithfully a report reproduces underlying session data) and nonmedical compliance (how successfully it avoids clinical register while remaining informative). RQ3 is therefore examined descriptively rather than hypothesis tested, and we treat its findings as feasibility evidence rather than as validation of a clinical reporting system.

### Goal of This Study

The goal of this feasibility and proof-of-concept study was therefore to determine whether passively collected physiological signals, behavioral observations, and semantic environment characteristics can support data-driven, between-session VR environment recommendation for residents with major neurocognitive conditions in long-term care and whether the resulting recommendations can be communicated transparently to families and care staff. Specifically, we tested 3 prospective hypotheses: that a model given environment features would use them to produce environment-specific recommendations whereas a physiology-only model would not (H1); that a hybrid behavioral-physiological target would differentiate between-environment effects better than a behavioral target alone (H2); and that water presence would be the strongest environment-level differentiator (H3). We additionally explored, descriptively, whether an LLM can generate evidence-traced, nonmedical reports from the pipeline’s outputs (RQ3). The study was designed to establish technical feasibility and to obtain a preliminary estimate of the environment-level signal; it was not designed to show that the recommendations improve resident outcomes, and no such claim is made. The report generation component is a prototype, and prospective validation against resident outcomes is identified as necessary future work.

## Methods

### Participants and Setting

Twenty-one residents of a long-term care facility in Québec, Canada, participated in this study. All had a documented diagnosis of a major neurocognitive condition. Demographic and clinical characteristics are summarized in Table 2. The mean age was 82.6 (SD 6.2; range 68‐97) years; age was missing for one participant. Twelve (57%) participants were female; sex was not documented for 1 (5%) participant. Cognitive diagnoses included Alzheimer disease (n=13, 62%), major neurocognitive condition of mixed or undetermined etiology (n=4, 19%), mixed dementia (n=2, 10%), and vascular dementia (n=1, 5%); the diagnosis was not documented for 1 (5%) participant. Functional status was assessed using the ISO-SMAF (ISO-classified Système de mesure de l’autonomie fonctionnelle [functional autonomy measurement system]) classification system [41] (mean profile 7.2, SD 0.7; range 7‐10; 1 value missing), indicating moderate-to-severe care needs. Most participants (n=18, 86%) could walk without aid; 2 (10%) used a walker; mobility was not documented for 1 participant. Seventeen (81%) participants wore corrective lenses, and 1 (5%) used a hearing aid. The hearing aid count reflects recorded hearing aid use rather than hearing status: hearing acuity was not formally assessed, and hearing aid use among long-term care residents is known to be low relative to the prevalence of hearing loss in this age group [42]. As the environments’ soundscapes were delivered through the headset’s audio, uncorrected hearing loss may have attenuated the audio dimensions of the environment encoding for some participants; this is noted in the Limitations section.

**Table 2.** Participant characteristics (N=21).

Characteristic	Value
Age^a^ (years), mean (SD; range)	82.6 (6.2; 68-97)
Female^a^, n (%)	12 (57)
Diagnosis, n (%)	
Alzheimer disease^a^	13 (62)
Major neurocognitive condition of mixed or undetermined etiology	4 (19)
Mixed dementia	2 (10)
Vascular dementia	1 (5)
Not documented	1 (5)
ISO-SMAF^b^ profile, mean (SD; range)	7.2 (0.7; 7-10)^a^
Corrective lenses, n (%)	17 (81)
Hearing aid, n (%)	1 (5)

a One value missing.

b ISO-classified Système de mesure de l’autonomie fonctionnelle (functional autonomy measurement system).

### Ethical Considerations

The study was approved by the Research Ethics Board (REB) of the Centre intégré universitaire de santé et de services sociaux (CIUSSS) de la Capitale-Nationale (protocol 2022‐2299, RIS_ForetStress) and conducted in accordance with institutional guidelines for research involving adults with cognitive impairment. As residents with major neurocognitive conditions cannot, in general, provide fully informed prospective consent, consent for participation followed the substitute decision-maker framework prescribed by institutional policy, with families notified through the facility’s standard communication channel. VR sessions were integrated into the facility’s existing leisure programming and delivered under the same care decision framework that governs other activities of daily living, rather than as a separate research-only procedure.

Per-session willingness was verified at each visit by a member of the recreation team together with the researcher, using ordinary, nontechnical language; residents who declined were not approached further, and sessions were terminated immediately at any sign of discomfort, agitation, or fatigue. Trained staff supervised all sessions and could terminate at any time. No monetary or material compensation was offered to participants or families: integration with the existing leisure programming was intended to remove any external incentive that might compete with a resident’s moment-to-moment willingness to participate.

All data were processed under coded participant identifiers (P01-P21); raw physiological recordings and individual-level behavioral ratings were retained within the institutional research infrastructure approved under the REB protocol, and no directly identifying information left that environment. Residual ethical questions arising from continuous physiological monitoring in a population that cannot fully articulate its preferences are discussed in the Limitations section.

### VR Sessions and Environments

Each participant received 2 immersive VR sessions per week over 10 consecutive weeks, yielding 420 total sessions (21 participants×20 sessions). Sessions were distributed across 5 virtual environments in a balanced rotation (84 sessions per environment): beach with kite surfers, Île d’Orléans countryside, forest and stream, Old Québec city walk, and active forest experience. Four environments were delivered using a white headset (Meta Quest 2; Meta Platforms; passive 360° video); one (active forest) used a blue headset (HTC Vive Cosmos; HTC Corporation) that supported a limited interactive element, specifically a trigger for forward advance; reverse movement was obtained by pivoting the resident’s chair. The blue headset is not an independent variable confounded with interactivity; it is the apparatus through which limited interaction was delivered, and the interaction demand dimension of the semantic encoding is designed to capture precisely this design variation. Interpretation of findings along the interaction demand dimension should nonetheless be made cautiously, because active forest is the only environment in the library with a nonzero interaction demand rating: any effect attributed to this feature in the importance analysis (reported in RQ2) is estimated from a single environment of contrast and cannot be distinguished from any other characteristic of active forest that the encoding did not capture. Water presence and social presence, which vary across the 4 white-headset environments, are not subject to this limitation. Sessions were supervised by trained staff who monitored the resident’s comfort and could terminate the session at any time. The mean recorded session duration was 11.4 (SD 3.0; range <1 to 25.7) minutes based on 371 sessions with wearable physiological recordings.

To characterize the perceptual properties of each VR environment, we developed a 10-dimension semantic encoding scheme covering visual complexity, motion (camera and scene), social presence, audio characteristics (nature and music), water presence, visual openness, interaction demand, and altitude. Two raters independently rated each environment on an ordinal 0 to 3 scale per dimension [43], normalized to [0, 1] for model input. Interrater reliability was handled via discussion-based reconciliation, given the small number of items (5 environments×10 dimensions). Full dimension definitions and rating rubrics are provided in Multimedia Appendix 1.

Independent prereconciliation ratings were not retained in machine-readable form, which precludes formal interrater reliability calculation (see Limitations section).

This encoding provides continuous, interpretable features that can mediate between-environment differences in physiological response and supports principled predictions for new environments not present during training (rate the new environment on the 10 dimensions and concatenate with physiological or behavioral inputs at inference time).

### Physiological Recording and Signal Processing

Physiological signals were recorded during each VR session using an EmotiBit wearable sensor (EmotiBit; Connected Future Labs) [44] attached to the participant’s forearm. The device captured PPG for cardiac signal derivation and EDA for sympathetic arousal monitoring. Of the 420 scheduled sessions, 374 (89%) yielded a matched physiological recording. Matching was performed automatically by aligning device time stamps with session scheduling logs, with manual verification for ambiguous cases.

Signals were processed through a dedicated quality control pipeline. Raw PPG recordings were segmented into analysis windows, and each window was evaluated for motion artifact, signal saturation, and interbeat interval plausibility. Windows failing quality criteria were excluded rather than reconstructed. From the usable windows, the pipeline extracted session-level summary features: mean HR, HRV (RMSSD, SDNN, and percentage of successive RR intervals differing by more than 50 ms [pNN50]), respiratory sinus arrhythmia (RSA) via Welch periodogram and Lomb-Scargle methods (RSA-HF), and window-level statistics (mean, median, SD, minimum, and maximum) for each base measure.

For PPG-derived features, 371 (99.2%) of 374 recorded sessions passed quality thresholds, with a mean usable window proportion of 87.7% (SD 22.6%). EDA processing used the configuration selected by the preprocessing search, with interpolation limited to gaps of ≤1 second: 218 (52%) of 420 sessions produced usable EDA, with a mean usable window proportion of 82.2% (SD 25.8%). From usable EDA segments, the pipeline extracted tonic skin conductance level (mean and SD) and phasic component features (root mean square amplitude and skin conductance response count).

The pipeline retained quality indicators as audit variables (eg, usable window percentage, motion artifact fraction, and device-algorithm HR disagreement) but did not include these as input features of the focal 23-feature model. Session duration, window counts, and the proportion of usable recording were preserved as session metadata and entered only the physiology-only feature set used for the ablation baseline (see Predictive Modeling section). Supplementary figures on study design, data quality, signal processing, and model diagnostics are provided in Multimedia Appendix 2. Full preprocessing parameters and quality control criteria are provided in Multimedia Appendix 1.

### Behavioral Measures

Two standardized instruments provided behavioral data for each participant.

#### Pittsburgh Agitation Scale

Trained observers completed the PAS-2 [45] after each VR session, rating 3 agitation domains: vocal intensity, motor agitation intensity, and aggressiveness intensity. Each domain was scored on a 5-point scale (0=absent to 4=severe). Ratings were available for 358 (85%) of 420 sessions; 62 sessions had missing PAS-2 data. As reported in the Descriptive Context section, the vocal intensity item was the primary behavioral input to the hybrid target definition: 234 (65.4%) rated sessions scored 0 (agitation absent), 116 (32.4%) scored 1 (low-volume, nondisruptive), and 8 (2.2%) scored 2 (moderately disruptive). No session reached levels 3 or 4.

#### Quality of Life in Late-Stage Dementia

The Quality of Life in Late-Stage Dementia (QUALID) [46] instrument was completed weekly by care staff who were familiar with the resident. It comprises 11 items capturing observable indicators of well-being: smile frequency, sadness, crying, facial distress, physical discomfort, vocal discomfort, aggressiveness, eating behavior, touch responsiveness, social engagement, and calmness. Items were scored on ordinal scales reflecting frequency over the preceding week. QUALID data were available for 400 (95%) of 420 sessions.

In the predictive model, QUALID items entered as input features (reflecting the resident’s recent behavioral context), while the PAS-2 vocal intensity item contributed to the target variable. This separation is intentional: QUALID describes the resident’s general state over the preceding week, whereas PAS-2 captures the immediate postsession observation. Using QUALID as an input allows the model to condition its predictions on the resident’s recent trajectory without circularly including the outcome measure as a predictor.

### Defining the Hybrid Calm Target

Standard behavioral agitation instruments capture observable distress but miss important ambiguities. A resident who appears behaviorally still may be physiologically aroused—withdrawn rather than comfortable. Conversely, a resident who vocalizes during a VR session may be physiologically relaxed—engaged rather than distressed. In our data, these ambiguities are compounded by a ceiling effect: 65.4% (234/358) of rated sessions already scored as behaviorally calm (PAS-2 vocal intensity score of 0), leaving limited room for between-environment differentiation using behavioral scores alone.

To address this, we defined a hybrid calm target that requires convergence of 2 independent signals: behavioral calm and elevated parasympathetic tone. A session is classified as calm in this operational sense (y=1) only if both conditions are met simultaneously: *y_hybrid*=1 if (PAS-2 vocal intensity=0) AND (RMSSD≥participant’s personal median RMSSD); *y_hybrid*=0 otherwise.

The behavioral component (a PAS-2 vocal score of 0) ensures the resident showed no observable vocal agitation. The physiological component (RMSSD at or above the participant’s own median) ensures the resident’s HRV was elevated relative to their personal baseline, indicating parasympathetic activation consistent with a relaxed autonomic state. The within-person median split accounts for the substantial between-person differences in resting autonomic tone that characterize this population [17]: what constitutes “elevated” RMSSD for one resident may be typical for another. Three further considerations motivated this choice over a fixed absolute threshold or a population cut point. First, with 6 to 20 usable sessions per participant, the median is the only within-person cut point that is robust to the skewed distribution of RMSSD without distributional assumptions. Second, a within-person split deliberately removes between-person differences in level from the physiological component of the target: person identity alone cannot predict whether a session falls above or below that person’s median, so any predictability of this component must come from within-person variation—including the environment presented—rather than from stable individual differences. Third, the same construction was applied uniformly to the RSA, RMSSD, and pNN50 variants evaluated in model selection, so that the variants differed only in the physiological index used. The within-person median was computed over all of a participant’s sessions, including those in the test period (weeks 8‐10), on the imputed and scaled RMSSD series; training labels therefore depend slightly on test period data, a point noted in the Limitations section.

This target is a constructed operational criterion, not an independently validated measure of well-being, and the term “calm” is used throughout as shorthand for meeting it. Two limitations follow directly from the median-split construction. By design, approximately half of each participant’s sessions satisfy the physiological component, whether or not the absolute within-person differences in RMSSD are large; and sessions whose RMSSD lies close to the participant’s median receive labels that are sensitive to measurement noise. Sensitivity analyses using alternative within-person thresholds and a continuous physiological component are reported in the Sensitivity of the Environment-Level Result section.

Within-person medians require an adequate number of RMSSD-bearing sessions per participant to be well defined. In this cohort, RMSSD was available for 369 (87.9%) of 420 sessions; per-participant counts ranged from 6 to 20 (mean 17.6, SD 3.3; median 19.0, IQR 16‐19). No participant fell below 5 RMSSD-bearing sessions, so within-person medians are defined for all 21 residents. Precision varies, however: for the most affected participant (P12, with 6 RMSSD-bearing sessions and 4 labeled sessions with observed RMSSD), the within-person median is more sensitive to individual observations than for participants with near-complete data, and this resident’s hybrid-target labels should be interpreted with correspondingly greater caution.

Three physiological variants of this hybrid target were tested during model selection: RSA via Welch periodogram (T5), RMSSD (T6), and pNN50 (T7). All 3 are time-domain or frequency-domain indicators of parasympathetic cardiac modulation. The selected model uses the RMSSD variant (T6). The choice among variants was determined empirically by validation performance; all 3 hybrid targets outperformed purely behavioral targets (PAS-2 vocal alone, summed agitation, and binary calm) in the automated search (see Predictive Modeling section).

Of 420 scheduled sessions, 358 (85.2%) carried a PAS-2 vocal intensity rating and received a hybrid-target label for supervised learning. For 35 of these, RMSSD was not observed and the physiological component was evaluated on the imputed value (the training period median), so their labels are less certain than the 323 (76.9%) based on observed RMSSD. The 62 (14.8%) sessions without a PAS-2 rating received a missing target mask: they were excluded from loss computation during training but retained in the input sequences to preserve each resident’s temporal context.

### Predictive Modeling

As defined in the Introduction, the modeling objective is environment-level discrimination: for each resident, the model must use environment features alongside physiological and behavioral inputs to rank the 5 VR environments by predicted probability of meeting the hybrid calm target. A model that captures only person-level effects, stable between-resident differences in baseline physiological and behavioral state, would not achieve this objective, even if its aggregate predictive accuracy were high. The physiology-only ablation model specified later in this section, together with the environment-permutation test described in the Evaluation Strategy section, is designed specifically to disentangle these 2 sources of variance.

The hypersearch evaluated 2108 configurations across 7 prediction targets, 6 feature sets, and 5 architectural families (long short-term memory, gated recurrent unit, lightweight transformer, ridge regression, and random forest; S1-S5 in Multimedia Appendix 1), each evaluated by 3-fold expanding-window cross-validation within the training period (weeks 1‐7); mean cross-validation mean absolute error (MAE) across folds was the ranking criterion. Training was not seeded, so individual fits are not reproducible bit for bit. The monitored cross-validation MAE averages the masked error over all time steps of the validation sequences, including training-week history cells, and therefore understates validation-week error; it is reported here as the search’s ranking score rather than as an estimate of out-of-sample error. The full grid specification is in Multimedia Appendix 1.

Models were trained on sessions from weeks 1 to 7 (294 sessions) and evaluated on sessions from weeks 8 to 10 (126 sessions, 6 per participant); MAE-based metrics use the 108 test sessions with a defined label, whereas the ranking metrics use all 126. Each participant’s sessions formed one chronological sequence with full history retained; the prediction for a given session uses that session’s own physiological, behavioral, and environment features together with all preceding sessions as context (causal attention), so the model estimates calm for the current session rather than forecasting it from past sessions alone. Environment recommendation is obtained by rescoring a session under each of the 5 environment encodings (counterfactual prediction), as shown in Figures 1 and 2. Within the training period, configurations were ranked by MAE across temporal cross-validation folds. The test set was reserved for final evaluation and was not used in the hypersearch ranking; its role in the choice of the focal configuration among the retained candidates is described below.

**Figure 1.**
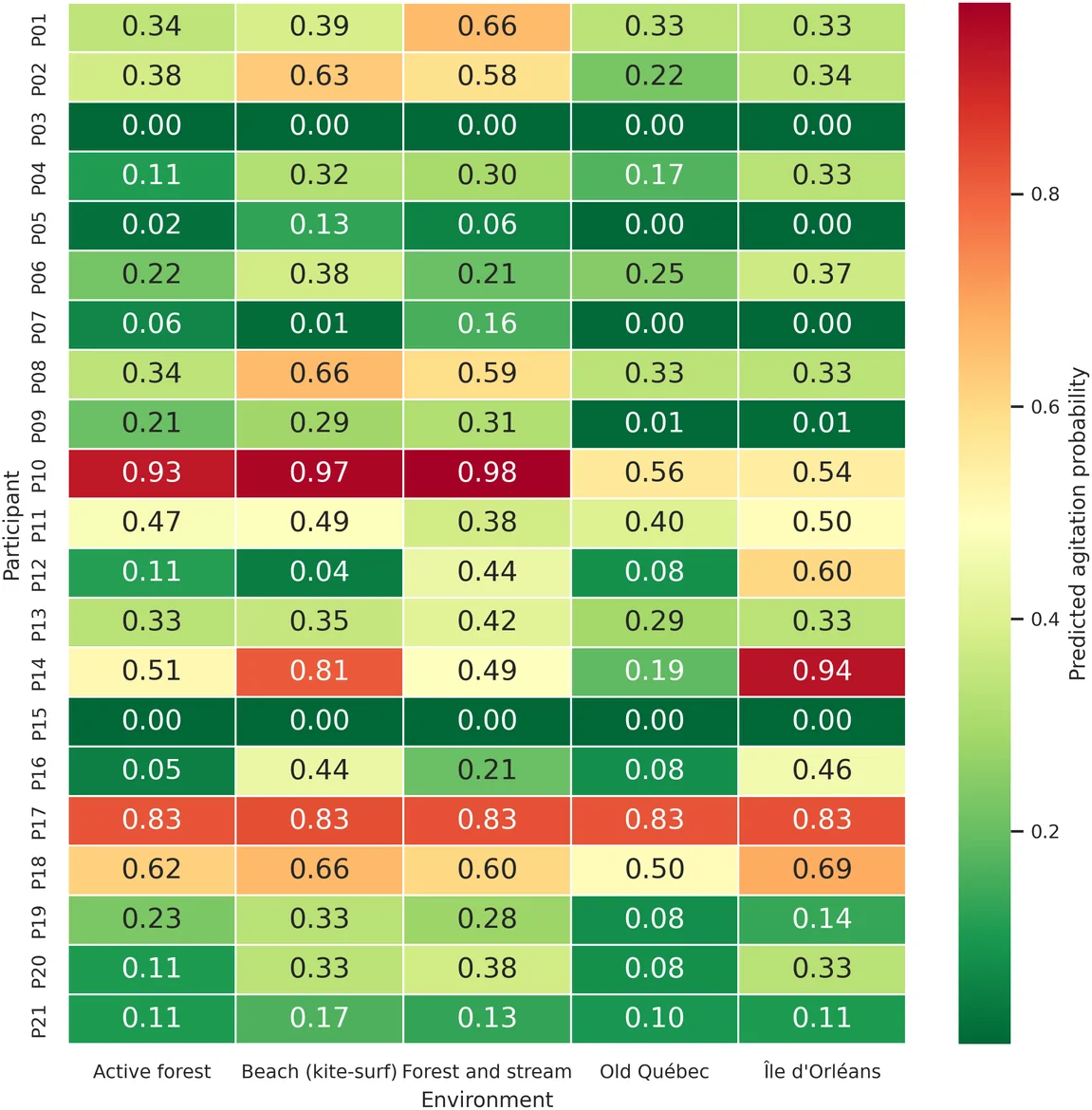
Recommendation heatmap for the 23-feature model. Each cell shows the mean predicted agitation probability for a given participant (row) in a given environment (column). Lower values (green) indicate calmer predicted sessions; higher values (red) indicate more agitated predictions. Between-participant variation dominates, but several participants show meaningful between-environment differentiation.

From the hypersearch, 5 environment-aware configurations and the physiology-only ablation (6 in total) were retained for full evaluation. Among the environment-aware feature sets, the best configuration for each (target, feature set) pair was identified and the 5 best of these representatives by cross-validation MAE were retained (test MAE 0.247‐0.304); the best physiology-only configuration was added as a no-environment ablation baseline (98 features: physiological summary statistics—PPG HR, HRV time domain, RSA spectral, EDA, and beat counts—and 4 session-level recording metadata features, with no environment or behavioral features). The ablation configuration was also the best configuration of the entire search by cross-validation MAE. The selected focal model uses 23 input features—10 physiological summary features (HRV time- and frequency-domain, EDA), 3 behavioral observation features (PAS-2 vocal intensity, QUALID smile, and QUALID calm or vocal discomfort), and 10 semantic environment features—implemented in TensorFlow 2.20 and Keras 3.10 (Google) as a small transformer [47] (1 encoder layer, hidden dimension 64, dropout 0.3, and learning rate 0.001) trained with binary cross-entropy on the hybrid RMSSD target (T6; see Defining the Hybrid Calm Target section). Three considerations entered the choice of this focal configuration among the retained candidates, and it is important to distinguish them. The first 2 are independent of the test set: the compact model’s cross-validation MAE (0.114) was within 0.02 of the other retained configurations (344‐368 features, 0.095‐0.111), although it was the lowest-ranked of the 5 on that criterion, and it was the only retained configuration compact enough for the per-feature importance analysis required by RQ2 to be interpretable (23 vs 344‐368 features); parsimony alone therefore singles it out, whereas cross-validation MAE alone would not. The third consideration was not independent of the test set: the environment permutation test (described in the Evaluation Strategy section) had been computed on the test set, and the finding that the compact model—unlike the larger configurations—retained environment sensitivity was known when the focal choice was fixed. The test-set environment permutation result for the focal model should therefore be read as a confirmatory test performed under a configuration whose selection was partly informed by that same test set, not as a fully independent replication; the Limitations section returns to this point. Larger configurations were retained as ablation baselines.

### Evaluation Strategy

The selected model and its ablation baseline were evaluated using 6 complementary approaches, each addressing a different aspect of model validity. Two sets of sensitivity analyses, added at revision in response to peer review and not prespecified, are described at the end of this subsection.

#### Environment Permutation Test

To determine whether the model actually uses environment information at inference time, we jointly shuffled the 10 environment feature columns across test sessions, disrupting the correspondence between each session’s physiological profile and its environment characteristics while preserving the marginal distribution of both. This procedure was repeated 200 times. The 1-sided P value was computed as (c+1)/(n+1), where c is the number of permutations in which the shuffled MAE was at or below the baseline MAE and n is the number of permutations; the same convention is used for all permutation tests reported below. A significant result (*P*<.05) indicates that the model’s predictions depend on environment features—that is, it has learned a person×environment interaction rather than simply predicting who is calm.

#### Feature Family Permutation Tests

The same shuffling procedure was applied separately to 3 feature families: all 10 physiological features, all 10 environment features, and all 3 behavioral (QUALID) features. Each family was shuffled 2000 times. This quantifies each family’s contribution to prediction accuracy. The environment family test parallels the environment permutation test above with an independent set of 2000 shuffles, so its estimate differs slightly; the additional tests provide context for interpreting the relative importance of physiology and behavioral observations.

#### Variance Decomposition

Test-set predictions were decomposed into participant, environment, and residual components using a 2-way ANOVA without interaction, computed with type III sums of squares. Type III was chosen to ensure that each effect estimate is independent of term-entry order in this unbalanced design (sessions are approximately but not exactly balanced across participants and environments after dropout). Both the sum-of-squares decomposition and the corresponding *F* tests are reported.

#### Ranking Metrics

For each participant, the model’s predicted calm probabilities were used to rank the available environments from most to least calming. These rankings were compared against the participant’s observed outcomes using 4 metrics: top-1 hit rate (proportion of sessions where the model’s top-ranked environment matched the observed calmest), top-2 and top-3 hit rates, pairwise accuracy (proportion of environment pairs correctly ordered), and mean Spearman ρ. As only 4 of 5 environments appeared in the test period (the beach environment was not scheduled during weeks 8‐10), all ranking evaluations are 4-way comparisons, with a chance baseline of approximately 25% for top-1 hit rate and 50% for pairwise accuracy. CIs (95%) were computed via 1000 bootstrap resamples of the test set [48].

#### Leave-One-Participant-Out Cross-Validation

To assess whether the model generalizes to residents not seen during training, we retrained the full pipeline 21 times, each time holding out all sessions from 1 participant. The held-out participant’s test period sessions were then predicted by a model that had never seen any of their data. Leave-one-participant-out (LOPO) MAE was computed per participant and summarized as a mean and SD across the 21 folds.

#### Report Evaluation

For each of 30 test period sessions (covering all 21 participants), the pipeline generated 2 plain-language reports: one for the resident’s family and one for clinical staff. Reports were generated by an LLM operating under a structured prompting framework that provided the model’s predictions, source physiological measurements, behavioral observations, and environment characteristics as input, along with constraints requiring nonmedical language, evidence tracing, and audience-appropriate tone. The report generation framework is described further in the RQ3 results.

Report quality was assessed on 9 dimensions: 2 binary compliance checks (medical language compliance [forbidden-term filter against a curated exclusion list of clinical or diagnostic terms] and length compliance [word count within the prescribed range]), 2 quantitative metrics computed against session ground truth (numerical fidelity [proportion of verifiable numerical claims matching session data] and evidence coverage [proportion of substantive claims with at least one cited evidence item]), and 5 qualitative ratings on a 1 to 5 Likert scale produced by a separate LLM serving as an automated grader (clarity, usefulness, actionability, fidelity, and nonmedical compliance). The use of an LLM-as-judge for assessing generated text quality—including chain-of-thought form-filling rubrics of the kind used here—is established methodology in natural language generation evaluation [49,50], with documented limitations (position, verbosity, and self-enhancement biases) that motivate the conservative interpretation of these ratings adopted in the RQ3 results. Detailed dimension definitions and rubric prompts are provided in Multimedia Appendix 1.

#### Sensitivity of the Target Definition (Added at Revision)

To examine how far the environment-level result depends on the median split construction of the hybrid target, the label was recomputed with the physiological component defined as RMSSD at or above the participant’s 40th percentile, 60th percentile, or upper tertile and as the median split with a 10% dead band that drops sessions within 10% of the participant’s median; agreement with the reported labeling was quantified by the number of changed labels and Cohen κ. The trained focal model’s test predictions were then held fixed and the environment permutation test (200 permutations, same shuffling scheme and random seed) was recomputed against each alternative labeling. Finally, the focal configuration was retrained on weeks 1 to 7 with a continuous target—the within-person z-score of session RMSSD, defined on PAS-2–calm sessions and fitted with a masked mean squared error loss—using 3 random seeds, and the environment permutation test was run on the 75 labeled calm test sessions. Thresholds and z-scores were computed over all of a participant’s sessions on the imputed series, as for the reported label.

#### Validation-Only Selection Criterion (Added at Revision)

To determine whether the focal configuration would have been selected without reference to the test set, the 6 retained configurations were retrained on the training period only (weeks 1‐7; 2 seeds each) using the pipeline’s 3-fold expanding-window cross-validation, and the environment permutation test (200 permutations) was computed on validation-week cells only. For each configuration, we report the monitored cross-validation MAE, the MAE on validation-week cells only, and the validation-fold environment permutation *P* value and effect size. The test set was not used in this analysis.

## Results

### Descriptive Context

Of 420 scheduled VR sessions, 358 (85.2%) received postsession PAS-2 ratings from trained observers. Among rated sessions, 234 (65.4%) scored 0 on the vocal intensity scale (agitation absent), 116 (32.4%) scored 1 (low-volume, nondisruptive vocalization), and 8 (2.2%) scored 2 (moderately disruptive). No session reached the 2 highest agitation levels (scores 3 or 4). Physiological recordings were obtained for 374 sessions (89%); of these, 371 passed PPG quality control, and 218 produced usable EDA. These figures confirm that immersive VR comfort sessions were generally well tolerated in this cohort. They also illustrate the ceiling effect that motivated the hybrid target: with nearly two-thirds of sessions already scoring as behaviorally calm, the PAS-2 vocal item alone provides limited between-environment differentiation.

### RQ1: Can the Model Identify Which Environment Calms a Given Resident?

#### Overview

This section tests hypothesis 1 (environment-level signal), which predicted that a model combining physiological, behavioral, and semantic environment features would use the environment features for differentiated recommendations, whereas a physiology-only model would not. The environment signal reached statistical significance, but the result is borderline. Jointly shuffling the 10 environment features across test sessions increased the model’s prediction error from a baseline MAE of 0.247 to a mean of 0.282 (SD 0.020) across 200 permutations; in 192 (96%) of 200 permutations, the shuffled MAE exceeded the baseline, and 8 were at or below it, yielding a 1-sided *P*=(8+1)/(200+1)=.045. The standardized distance from the baseline to the permutation distribution was large—Cohen *d*=1.75, computed as the difference between the mean shuffled MAE and the baseline MAE, divided by the SD of the shuffled MAE distribution. Repeating the test with 2000 permutations—keeping the trained model, baseline MAE, and shuffling scheme unchanged—yielded *P*=.04 (*d*=1.76), confirming that the estimate is stable under higher Monte Carlo iteration counts and narrowing the estimate’s sampling error from approximately 0.015 to 0.004. This is consistent with the model using environment information at inference time: disrupting the correspondence between a session’s physiological context and its environment characteristics measurably degrades predictions. Given the borderline *P* value, the single configuration tested, and the selection considerations described in the Methods section, we regard this result as preliminary evidence that requires independent replication.

This finding was specific to the compact, 23-feature model. The 4 other environment-aware configurations tested—all using 344 to 368 features—yielded a *P* value of >.79 on the same environment permutation test (*P*=.80‐.97; the configurations are those listed in Table 3). In those models, shuffling environment features either had no effect or slightly improved predictions, indicating that the models had learned to ignore environment entirely. With hundreds of features available, including physiological summary statistics and their associated missingness indicators, those models found sufficient information to predict who is calm without attending to which environment was presented. Only when the feature set was constrained to 23 features—10 physiological, 3 behavioral, and 10 environment—was the model forced to use every available signal, including environment. This property was not evident on validation folds when the same configuration was retrained on the training period only (see Sensitivity of the Environment-Level Result section); it therefore characterizes the particular trained model rather than the configuration.

**Table 3.** Validation-only selection criterion.^a^

Configuration	Target	Feature set (n)	Monitored CV^b^ MAE^c^ (stored)	Validation-week MAE (seed 42/1337)	Validation environment permutation *P* (seed 42/1337)	*d* (seed 42/1337)
Rank 1	T5 (RSA^d^, Welch)	All except QUALID^e^ (344)	0.095	0.285/0.297	.15/.19	1.04/0.86
Rank 2	T7 (pNN50^f^)	All (368)	0.108	0.304/0.315	.44/.70	0.18/−0.51
Rank 3	T5 (RSA, Welch)	All (368)	0.109	0.312/0.302	.52/.26	−0.13/0.68
Rank 4	T7 (pNN50)	All except QUALID (344)	0.111	0.292/0.301	.55/.13	−0.17/1.24
Rank 5 (focal, reported)	T6 (RMSSD^g^)	Reduced (23)	0.114	0.274/0.281	.83/.80	−1.02/−0.80
Ablation	T6 (RMSSD)	Physiology only (98)	0.089	0.247/0.246	—^h^	—

a The 6 retained configurations were retrained on weeks 1‐7 only (2 seeds each) with the pipeline’s 3-fold expanding-window cross-validation (CV); the environment permutation test (200 permutations) was computed on validation-week cells only, with the test set untouched. Monitored CV MAE (mean absolute error) is the search’s ranking score for the stored configuration (see Methods); validation-week MAE is computed on validation-week cells only.

b CV: cross-validation.

c MAE: mean absolute error.

d RSA: respiratory sinus arrhythmia.

e QUALID: Quality of Life in Late-Stage Dementia.

f pNN50: percentage of successive RR intervals differing by more than 50 ms.

g RMSSD: root mean square of successive differences.

h Not applicable.

As a complementary check, we ran a label permutation test: the hybrid target was randomly reassigned across sessions, with a fresh model retrained on each of 30 permutations, and the distribution of resulting test MAEs was compared to the real model’s. This yielded a *P* value of .45—the real model’s overall predictive accuracy does not significantly exceed what can be achieved by fitting on shuffled targets. The environment permutation test and the label permutation test answer different questions and are both informative. The environment test (*P*=.045) asks whether the model uses environment features at inference time; the label test (*P*=.45) asks whether aggregate predictive accuracy exceeds baseline structure in the inputs. A significant environment permutation result alongside a null label permutation result is consistent with a small effect: the model uses environment information detectably, but the resulting accuracy gain is small enough that overall MAE is dominated by person-level structure already present in the inputs.

#### Person-Level Effects Dominate; Environment-Level Discrimination Is Modest

A type III 2-way ANOVA on the model’s test-set predictions confirmed a strong participant main effect (*F*_20,84_=2.98; *P*<.001; partial *η*²=0.415) and no detectable environment main effect (*F*_3,84_=0.10; *P*=.96; partial *η*²=0.004). The large participant component confirms that the strongest predictor of a calm session is who the resident is—their baseline autonomic profile, behavioral history, and overall responsiveness. The environment main effect is not resolvable by marginal ANOVA on aggregate test-set predictions; its presence is established instead by the permutation test reported earlier, which is sensitive to whether the model uses environment information at inference time, even when aggregate between-environment mean differences are small.

Ranking metrics on the test set (4 environments, 126 sessions) are presented with 95% bootstrap CIs (1000 resamples). The model correctly identified the calmest environment for a given participant’s session 27.8% (35/126) of the time (top-1 hit rate; 95% CI 19.8%‐35.7%; chance ≈25%). The correct environment fell within the model’s top 2 recommendations 61.1% (77/126) of the time (95% CI 52.4%‐69.8%) and within the top 3 recommendations 84.9% (107/126) of the time (95% CI 78.6%‐91.3%). Pairwise accuracy—the proportion of environment pairs correctly ordered—was 57.9% (chance 50%). The mean Spearman ρ between predicted and observed environment rankings was 0.27 (95% CI 0.17‐0.36).

The top-1 CI includes the chance baseline. This is not a contradiction: the rank metric and the permutation test ask different questions. The rank metric (top-1 hit rate) asks whether the model’s single top recommendation matches the empirically calmest environment at above-chance frequency—a demanding point estimate test. The permutation test asks whether the model uses environment information at all—a direction of signal test. A significant permutation test alongside a rank-metric CI that spans chance is internally consistent: a signal is detectable, but it is too small to produce reliably correct top 1 rankings at the present sample size. The evidence for environment use therefore rests on the permutation test (*P*=.045) and on the qualitative pattern of predictions described next, and its practical value for recommendation remains to be established.

#### The Model Produces Differentiated Environment Recommendations

Figure 1 shows the recommendation heatmap: for each participant, the model’s mean predicted agitation probability across all 5 environments (including counterfactual predictions for environments not experienced in a given test session). Several patterns are evident.

First, some residents show strong between-environment differentiation. Participant P10, for instance, received predicted agitation probabilities of 0.56 for Old Québec and 0.54 for Île d’Orléans, compared with 0.93 for active forest and 0.98 for forest and stream—a clear preference for the 2 culturally grounded, lower-nature environments. Participant P14 showed a contrasting profile: calmest in Old Québec (0.19) but most agitated in Île d’Orléans (0.94) and beach (0.81), despite P10 and P14 sharing Old Québec as a preferred environment. Participant P09 showed particularly strong differentiation, with predicted probabilities near zero for Old Québec (0.01) and Île d’Orléans (0.01) but 0.31 for forest and stream.

Second, some residents show flat profiles. Participant P03 received predictions near 0.00 across all 5 environments—consistently calm regardless of content. Participant P17 showed the opposite pattern: approximately 0.83 everywhere (consistently agitated). The model correctly identifies these residents as environment insensitive rather than forcing a spurious preference.

Third, the variation is continuous rather than binary. Most participants fall between the extremes of P03 and P17, with moderate differentiation that varies in magnitude and direction across environments. This continuous variation is consistent with the clinical reality that environment preferences are subtle rather than categorical.

#### Without Environment Features, Predictions Are Accurate but Undifferentiated

The physiology-only ablation model achieved a test MAE of 0.258 for the retrained model whose predictions are analyzed here (0.220 at the hypersearch evaluation stage)—comparable to the 23-feature model’s 0.247—consistent with physiology alone being a strong predictor of who will be calm. However, the ablation model’s environment permutation test yielded a *P* value of >.99 by definition: with zero environment features, there is nothing to shuffle. More importantly, the ablation model’s predictions are nearly identical across environments for each participant. Where the 23-feature model predicted agitation probabilities ranging from 0.19 to 0.94 across environments for P14, the ablation model predicted a single value of 0.05 across all 5 environments—effectively a flat line.

Figure 2 shows the key contrast: side-by-side heatmaps for the 23-feature model and the ablation. The 23-feature model produces visible between-environment variation for most participants; the ablation produces horizontal bands—each participant has a characteristic prediction level, but that level does not change across environments. The ablation tells us who is calm. The 23-feature model additionally tells us which environment calms them.

**Figure 2.**
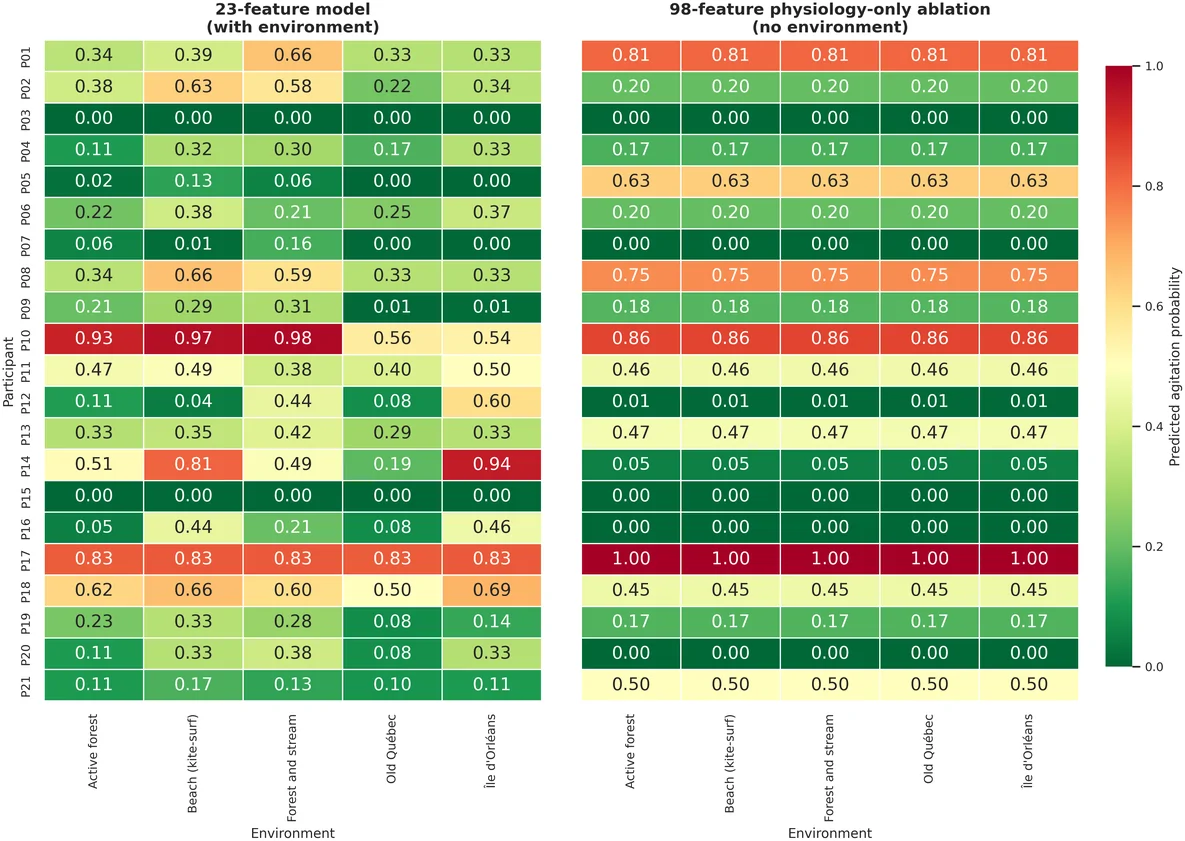
Side-by-side comparison of environment recommendation heatmaps: the 23-feature model (left) versus the physiology-only ablation model (right). Each row represents 1 participant (21 total); each column represents 1 of the 5 virtual reality environments. Cell color encodes the model’s mean predicted agitation probability for that participant-environment pair—green for calmer predictions and red for more agitated; the color scale is shared between the 2 panels to allow direct visual comparison. The takeaway is the horizontal texture of each panel. In the 23-feature model (left), most rows show visible across-environment color variation, indicating that the model’s prediction for a given resident depends on which environment is presented. In the ablation (right), rows appear as near-uniform bands: the model predicts approximately the same agitation probability for each resident regardless of environment, reflecting that it has learned participant-level rather than environment-level structure. This visual contrast is the qualitative counterpart to the quantitative environment-permutation test (reported in research question 1: *P*=.045 for the 23-feature model; *P*>.99 by definition for the ablation, which contains no environment features to shuffle).

#### Generalization to Unseen Participants

LOPO cross-validation yielded a mean MAE of 0.349 (SD 0.237) across 21 folds. The high variance reflects the person-dominated nature of the data: some participants are trivially predictable from the patterns of the remaining 20 (P03: MAE≈0.00; P07: MAE≈0.00—both consistently calm), while others’ response profiles are not well captured by the group model (P15: MAE≈1.00; P08: MAE=0.74). This variability is consistent with the dominant participant effect observed in the type III ANOVA (partial η²=0.42) and does not indicate model instability—rather, it highlights that individual response profiles in this population are genuinely heterogeneous.

Taken together, these results are consistent with hypothesis 1: the 23-feature model uses environment information for recommendations (environment permutation *P*=.045), whereas the physiology-only ablation does not and produces undifferentiated predictions consistent with person-level rather than environment-level discrimination. This support is qualified by the borderline *P* value, by its derivation from a single selected configuration, and by a top-1 CI that includes chance.

### RQ2: Which Features Contribute Most to Environment Recommendation?

This section tests hypothesis 2 (hybrid target) via the model search results described in the Predictive Modeling section and feature-family permutation importance reported below, and tests hypothesis 3 (water presence) via the individual-feature permutation importance in Table 4.

**Table 4.** Permutation importance for all 23 input features (30 repeats per feature).

Rank	Feature	Family	ΔMAE^a^
1	RSA^b^ (HF^c^ Welch, session)	Physiology	+0.095^d^
2	RMSSD^e^ mean	Physiology	+0.040
3	QUALID^f^: calm	Behavioral	+0.038
4	QUALID: vocal discomfort	Behavioral	+0.035
5	Water presence	Environment^g^	+0.028
6	QUALID: smile	Behavioral	+0.028
7	pNN50^h^ mean	Physiology	+0.017
8	SDNN^i^ mean	Physiology	+0.013
9	EDA^j^ tonic mean	Physiology	+0.007
10	RSA (HF Lomb, session)	Physiology	+0.002
11	Interaction demand	Environment^g^	+0.001
12	Social presence	Environment^g^	+0.001
13	EDA phasic RMS^k^	Physiology	+0.001
14	Visual complexity	Environment	+0.001
15	Camera motion	Environment	<0.001
16	HR^l^ mean	Physiology	<0.001
17	Visual openness	Environment	<−0.001^m^
18	Scene motion	Environment	−0.001
19	EDA tonic SD	Physiology	−0.001
20	HR variability (SD)	Physiology	−0.002
21	Altitude	Environment	−0.003
22	Audio: music	Environment	−0.007
23	Audio: nature	Environment	−0.011

a MAE: mean absolute error.

b RSA: respiratory sinus arrhythmia.

c HF: high frequency.

d Positive ΔMAE: shuffling the feature increases prediction error (feature is useful).

e RMSSD: root mean square of successive differences.

f QUALID: Quality of Life in Late-Stage Dementia.

g Environment features are highlighted where importance is positive and interpretable.

h pNN50: percentage of successive RR intervals differing by more than 50 ms.

i SDNN: SD of normal-to-normal RR intervals.

j EDA: electrodermal activity.

k RMS: root mean square.

l HR: heart rate.

m Negative ΔMAE: shuffling improves predictions (feature carries noise).

#### All Three Feature Families Contribute Significantly, With Physiology Dominant

Family-level permutation tests—jointly shuffling all features within a family and measuring the increase in test MAE—confirmed that physiology, environment, and behavioral features each carry information the model uses. Across 2000 permutations per family, shuffling the 10 physiological features produced the largest degradation (mean ΔMAE 0.128, SD 0.028; *P*<.001), followed by the 10 environment features (mean ΔMAE 0.047, SD 0.020; *P*=.02) and the 3 QUALID features (mean ΔMAE 0.046, SD 0.021; *P*=.01). The physiological contribution is approximately 3 times larger than either of the other 2 families, consistent with the ANOVA finding that person-level physiology dominates predictions. However, the environment and behavioral families are both independently significant: removing either one degrades performance beyond what would be expected by chance.

This result, together with the model-search finding earlier—that all 3 hybrid-target variants (RSA, RMSSD, and pNN50) outperformed every purely behavioral target in the 2108-configuration search—is consistent with hypothesis 2. The hybrid target was necessary in the sense that purely behavioral targets could not reach the top of the selection and sufficient in the sense that the selected hybrid target yielded an environment-aware model in which all 3 feature families contribute independently; whether it captures well-being as such is a separate question addressed in the Limitations section.

#### Water Presence Is the Most Influential Individual Environment Feature

Table 4 presents the full 23-feature permutation importance ranking. Among environment features, water presence ranked fifth overall (ΔMAE +0.028), above 1 of the 3 behavioral features and most physiological features. To put this in context, shuffling water presence alone increases prediction error by approximately 30% of the combined individual importance of the 3 QUALID items. Interaction demand ranked 11th (ΔMAE=+0.001) and social presence ranked 12th (ΔMAE=+0.001)—both positive but small. These 3 features—water, interaction demand, and social presence—are the environment dimensions along which the 5 VR environments differ most (Table 1), suggesting the model has learned to exploit the contrasts that are actually present in the data. This result is consistent with H3: among the semantic environment features, water presence is indeed the strongest differentiator of between-environment calm, consistent with the blue-space literature reviewed in the Introduction.

#### Some Environment Features Show Negative Importance

Audio nature (ΔMAE=−0.011) and audio music (ΔMAE=−0.007) showed negative permutation importance: shuffling them slightly improved predictions. This indicates that the model learned weak associations with these dimensions that do not generalize to the test set—plausibly because audio music is confounded with a single environment (Île d’Orléans is the only environment with music) and audio nature has limited contrast across the remaining environments. These negative-importance features are reported transparently. The joint 10-feature environment permutation test (*P*=.045) indicates a net environment signal despite noise in individual features, subject to the caveats already noted.

### Sensitivity of the Environment-Level Result

Two sets of analyses, added at revision in response to peer review and not prespecified, examined how far the environment-level result depends on the definition of the target and on the way the focal configuration was selected (see Evaluation Strategy section).

The hybrid label was robust to the threshold. Alternative within-person cut points (40th percentile, 60th percentile, and upper tertile) changed 19 to 31 of the 358 labels (κ=0.79‐0.89 relative to the median split), and a 10% dead band around the median removed 98 near-median sessions without changing any retained label (Table 5). The environment permutation result was not robust to the same changes. Rescoring the trained model’s unchanged test predictions against each alternative labeling, shuffling the environment features still increased the error in every variant except the dead-band one (*d*=0.93‐1.37), but none reached significance (*P*=.10‐.20; dead band P=.57; *d*=−0.13). Retraining the focal configuration with a continuous target—within-person z-scored session RMSSD on PAS-2-calm sessions—gave the same picture across 3 seeds: environment shuffling increased the error (*d*=0.65‐1.60), with *P*=.06, .23, and .25 (Table 5). The direction of the effect was therefore consistent, but its statistical significance was specific to the median-split labeling of the single trained model reported under RQ1.

**Table 5.** Sensitivity of the environment permutation test to the definition of the hybrid calm target.^a^

Target labeling	Positive rate	κ vs reported labeling	Test sessions (n)	Test MAE^b^	Mean shuffled MAE	*P* value (*d*)
RMSSD^c^ ≥ within-person median (reported)	0.34	1	108	0.247	0.282	.045 (1.75)
RMSSD ≥40th percentile	0.39	0.89	108	0.268	0.293	.10 (1.37)
RMSSD ≥60th percentile	0.27	0.84	108	0.219	0.239	.18 (0.98)
RMSSD in upper tertile	0.25	0.79	108	0.232	0.250	.20 (0.93)
Median with 10% dead band^d^	0.32	1	75	0.211	0.209	.57 (−0.13)
Continuous *z*-scored RMSSD, calm sessions, retrained						
Seed 42^e^	—	—	75	0.306	0.317	.06 (1.60)
Seed 1337^e^	—	—	75	0.338	0.345	.23 (0.83)
Seed 2026^e^	—	—	75	0.310	0.315	.25 (0.65)

a The first 5 rows rescore the trained focal model’s unchanged test predictions against alternative labelings; the last 3 rows retrain the focal configuration with a continuous target (3 seeds). *P* values use the (c+1)/(n+1) convention with 200 permutations; *d* is the standardized distance between the baseline MAE and the shuffled distribution.

b MAE: mean absolute error.

c RMSSD: root mean square of successive differences.

d The dead band drops sessions rather than relabeling them; κ is computed on the 260 retained sessions.

e MAE in *z* score units, not comparable with the binary-label MAE of the first five rows; the positive rate and κ do not apply to a continuous target (234 calm sessions with a defined target).

A selection criterion computed on training period data only did not select the compact model. When the 5 retained configurations were retrained on weeks 1 to 7 and the environment permutation test was computed on validation-week cells, no configuration showed significant environment sensitivity, and the compact model showed the least: shuffling its environment features slightly improved its validation error (*P*=.80‐.83; *d*=−1.02 and −0.80), whereas the 344-feature rank-1 configuration came closest to significance (*P*=.15‐.19; *d*=1.04 and 0.86; Table 3). By monitored cross-validation MAE alone, the criterion would have selected the physiology-only configuration and, among environment-aware configurations, the 344-feature rank-1 configuration. The validation-week-only MAE (0.25‐0.32 for every configuration) also confirms that the monitored cross-validation scores used to rank the search (0.09‐0.11) understate out-of-sample error, as noted in the Methods section.

Taken together, the environment-level signal reported under RQ1 should be read as exploratory. Its direction was consistent across target definitions and seeds, but its statistical significance was not reproducible under alternative labelings, and the property that motivated the focal choice—sensitivity to environment features—was not visible on training period data. Two limitations of these analyses are noted: the threshold analysis rescored a model trained on the median-split labels rather than retraining per threshold, which may penalize the alternative labelings; and the retraining analyses used 2 or 3 seeds of an unseeded pipeline, so their *P* values are indicative rather than precise.

### RQ3: Can an LLM Generate Evidence-Traced, Nonmedical Reports?

#### Overview

RQ3 is examined as an exploratory feasibility check on the report-generation component of the pipeline. The analyses below describe what the component produces (compliance, numerical fidelity, and evidence coverage) and how an automated judge rates the output. The evaluation is deliberately scope limited: it uses 60 reports generated from the 30 test period sessions, assessed by a second LLM rather than by care staff or family members, and it does not test whether the reports change decisions or improve care processes. Clinical validity—whether reports are useful, safe, and acceptable in actual practice—requires prospective human evaluation and is identified as necessary future work (see Limitations section).

#### Report Generation and Scope

For 30 test period sessions covering all 21 participants, the pipeline generated 2 plain-language reports for each session: 1 addressed to the resident’s family and 1 to clinical staff, yielding 60 reports in total. Each report was produced by an LLM (Claude Sonnet 4; Anthropic) operating under a structured prompting framework. The prompt provided the model’s predictions, the session’s physiological measurements, the observer’s behavioral ratings, the environment’s semantic characteristics, and the resident’s recent session history as input. Explicit constraints required nonmedical language, evidence tracing (every factual claim linked to a source data point), and audience-appropriate tone and detail level (Figure 3).

**Figure 3.**
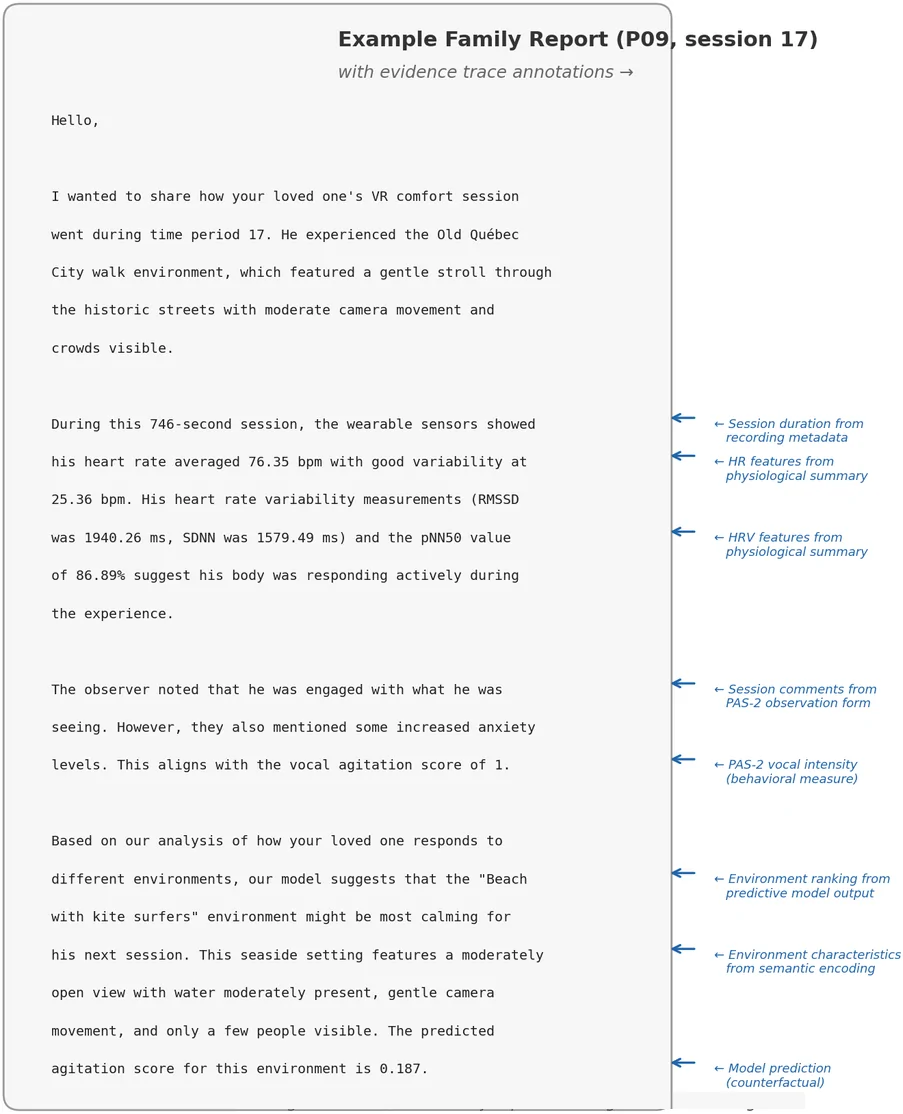
Annotated example of a family-audience report, illustrating the evidence-tracing framework that links every factual claim in generated prose back to a source data point. The example shown is drawn from participant P09, session 17. Blue overlay annotations mark each traced claim and label its provenance—physiological measurement, behavioral observation, model prediction, or session metadata. Reader takeaways: (1) the report is written in plain, nonmedical language appropriate for a family audience; (2) every numeric value and qualitative descriptor that appears in the prose is anchored to specific input data points rather than generated free-form; (3) modeled quantities (predicted probabilities and recommended environment rankings) are distinguished from observed quantities (measured heart rate variability [HRV] and rated agitation) in the annotation scheme. The framework is intended to make automated reports auditable by care staff or family members who cannot inspect the underlying model directly; its limits, including the fidelity-compliance trade-off documented in the research question 3 results, are discussed in the Discussion. HR: heart rate; PAS-2: Pittsburgh Agitation Scale, second version; pNN50: percentage of successive RR intervals differing by more than 50 ms; RMSSD: root mean square of successive differences; SDNN: SD of normal-to-normal RR intervals; VR: virtual reality.

#### Safety and Compliance

All 60 reports passed both binary compliance checks: zero instances of forbidden medical terminology (100% compliant) and all reports within prescribed length limits (family: mean 330, SD 17, range 294‐368 words; staff: mean 468, SD 29, range 415‐520 words).

#### Accuracy Metrics

Numerical fidelity—the proportion of verifiable numbers in each report that matched the source evidence data—averaged 95.4% (SD 9.2%) across all reports. Evidence coverage—the proportion of key evidence items referenced in each report—averaged 89.5% (SD 11.6%).

#### Automated Quality Ratings

A second LLM (Claude Opus 4; Anthropic), serving as an automated judge, rated each report on 5 dimensions (1‐5 Likert scale). These ratings are reported as exploratory automated measures rather than as validated indicators of report quality: LLM-as-judge outputs correlate imperfectly with human judgment and carry known limitations in clinical and clinical-adjacent text evaluation [33]. Human evaluation by actual care staff and family members is identified as necessary future work (see Limitations section). Across all 60 reports, mean automated-judge ratings of clarity, usefulness, actionability, fidelity, and nonmedical compliance were 4.08 (SD 0.28), 3.97 (SD 0.32), 3.92 (SD 0.38), 4.23 (SD 0.83), and 3.60 (SD 0.98), respectively.

#### The Fidelity-Compliance Trade-Off

The most notable finding from the report evaluation was a systematic difference between the 2 audience types (Table 6). Staff reports achieved substantially higher numerical fidelity (98.5% vs 92.3%) and evidence coverage (97.8% vs 81.3%) than family reports. They also received higher fidelity ratings from the automated judge (4.70 vs 3.77). However, staff reports scored markedly lower on the nonmedical compliance Likert dimension (2.83 vs 4.37). This dimension operationalizes register-level markers that the binary forbidden-terms filter does not capture—diagnostic framing, prescriptive phrasing, medical jargon beyond the forbidden list, and clinical documentation tone. Its divergence from the 100% forbidden-terms compliance rate indicates that the language model avoided the prohibited vocabulary yet drifted into a clinical register when asked to produce data-rich, precise summaries for a professional audience.

**Table 6.** Report evaluation by audience type.^a^

Metric, mean (SD)	Family (n=30)	Staff (n=30)
Numerical fidelity (%)	92.3 (12.1)	98.5 (2.5)
Evidence coverage (%)	81.3 (10.8)	97.8 (4.2)
Clarity (1-5)	4.17 (0.38)	4.00 (0.00)
Usefulness (1-5)	4.00 (0.26)	3.93 (0.37)
Actionability (1-5)	4.03 (0.32)	3.80 (0.41)
Fidelity (1-5)	3.77 (0.82)	4.70 (0.53)
Nonmedical compliance (1-5)	4.37 (0.56)	2.83 (0.65)

a Numerical fidelity and evidence coverage are programmatic accuracy metrics computed against source evidence. Likert ratings (clarity, usefulness, actionability, fidelity, and nonmedical compliance) are exploratory automated scores produced by a second large language model serving as a judge, not validated quality indicators; human evaluation is planned as future work. Values are mean (SD) over 30 reports per audience.

Family reports showed the inverse pattern. The language model simplified data for accessibility, producing warmer and more clearly nonmedical prose, but at the cost of some numerical precision and evidence coverage. Family reports scored higher on actionability (4.03 vs 3.80), suggesting that the simplified framing made recommendations more concrete and implementable for a lay audience.

This pattern is not a failure of the prompting framework; it reflects a genuine tension in clinical communication. Maximizing fidelity to source data pulls language toward technical precision; maximizing nonmedical accessibility pulls it toward narrative simplification. The 2 objectives are partially in conflict, and the language model navigates this trade-off differently depending on the target audience. Future iterations of the prompting framework should explore whether this gap can be narrowed, and human evaluation by actual care staff and families is needed to determine which balance point is preferred in practice.

## Discussion

### Principal Findings

This study set out to determine whether a data-driven system could identify which VR environment produces the calmest experience for a given resident with a major neurocognitive condition, using wearable physiology, behavioral observations, and semantic environment descriptors. Three findings ground the contribution.

First, an environment-level signal is statistically detectable from the available data. The compact 23-feature model showed a significant drop in prediction accuracy when environment features were permuted (*P*=.045; Cohen *d*=1.75), while the 4 larger environment-aware configurations (344 to 368 features) ignored environment entirely (all *P*>.79). The signal is small—person-level variance dominated at partial η²=0.42—and borderline in statistical terms, and it was recovered only when the feature set was constrained to keep environment information from being buried by physiology and missingness patterns. As the focal configuration was selected in part with knowledge of this test-set result and because sensitivity analyses showed the significance to be specific to the median-split labeling while the direction of the effect was consistent, the finding is exploratory and requires independent replication.

Second, the hybrid behavioral-physiological target, as operationalized here, was both necessary and sufficient for that signal to emerge. No purely behavioral target was among the 5 configurations retained from more than 2100 evaluated: with 65% of sessions already rated as behaviorally calm, PAS-2 vocal intensity alone has too little variance to differentiate environments. Among the environment dimensions, water presence carried the most weight, ranking first among the 10 environment features and fifth overall.

Third, automated plain language reporting from the model’s outputs is technically feasible, with a documented audience-specific trade-off: staff-targeted reports achieved higher fidelity ratings (4.70/5) but drifted toward clinical register (compliance 2.83/5), while family-targeted reports stayed firmly nonmedical (4.37/5) but sacrificed precision (3.77/5). All 60 generated reports passed binary safety checks.

Two distinct claims warrant separation. The first—that the model uses environment information at inference time, in a person-specific way, and that its outputs can be surfaced in plain-language reports—is what the primary analyses suggest, with the qualifications documented in the sensitivity analyses. The second—that the rankings the model produces are clinically optimal, in the sense that the environment placed first for a given resident is in fact the one most likely to benefit them in routine care—is not and would require prospective validation against in-session distress outcomes in a held-out cohort. We frame the present results as motivation and effect-size estimate for such a validation study, not as evidence that the system is ready to inform care decisions.

### Comparison With Prior Work

The recommendation pipeline reported here occupies an operational niche left open by 4 adjacent literatures. Within VR personalization specifically, the systematic review by Li et al [13] of 132 studies cataloged real-time adaptation in cognitively intact users, with most systems operating during a session via EEG, GSR or EDA, or HR signals. Between-session environment selection from a curated library—what the present pipeline does—was not an explicit category in their taxonomy, and residential long-term care for residents with major neurocognitive conditions was not a represented application domain. The contribution here is therefore at a complementary timescale and population to the dominant real-time adaptation paradigm.

Within wearable-physiology work in dementia care, Iaboni et al [7] established that person-specific multimodal models can classify behavioral symptoms substantially better than population-level models. The present pipeline extends that finding in 2 directions: it adds semantic environment features to the same physiological-modeling pattern, and it shifts the prediction target from agitation detection (“Is this resident agitated now?”) to environment selection (“Which environment is most likely to keep them calm next session?”). The dominance of person-level variance reported here (partial η²=0.42) is consistent with the person-specific result of Iaboni et al. What is added is preliminary evidence that, layered on top of person-specific physiology, a smaller environment-level signal may be recoverable when feature parsimony preserves it—a methodological point with practical consequences: in applied dementia care machine learning, exhaustive feature extraction can bury the signal of interest, and the choice of feature-set size is itself an RQ-aligned design decision rather than a regularization detail.

Within the tailoring tradition in dementia care, Cohen-Mansfield et al [11] and the Tailored Activity Program of Gitlin et al [12] established that individualized nonpharmacological interventions, matched to each resident’s unmet needs or preferences, improve agitation and functional outcomes. Both relied on human-administered tailoring at the authoring or delivery stage. The present pipeline contributes a data-driven environment recommendation mechanism in that lineage—supporting between-session decisions by care staff with a resident’s own session-to-session response data, rather than executing within-session content adaptation. The hybrid behavioral-physiological target used here extends naturally to other structured activities in dementia care where observer ratings are available alongside physiological recording—music therapy, multisensory stimulation, and outdoor walks—in which neither channel alone is sufficient to define comfort [7,18].

Finally, the prominence of water presence among environment features replicates, at the feature importance level, the result of Tanja-Dijkstra et al [40], who found that VR beach walks produced greater anxiety reductions than VR city walks in a randomized trial. The 3 environments with the highest water-presence ratings (forest and stream 1.0, beach 0.67, and Île d’Orléans 0.33, tied with active forest) showed the most differentiated calm responses across residents here. The convergence of these 2 independently obtained results—a randomized between-environment effect in cognitively intact users, and a feature-importance ranking in residents with major neurocognitive conditions—is consistent with the possibility that the blue-space exposure literature [38,39] transfers to immersive VR in dementia care, although the present feature-importance result is preliminary. Social presence and interaction demand also contributed positively, suggesting that the social and interactive character of a virtual scene carries person-specific information; for content developers, a tentative implication is that water elements, social density, and interactivity level deserve attention as design variables when curating VR libraries for this population. The audience-specific trade-off in automated reporting—fidelity preserved for staff at the cost of clinical drift, nonmedical register preserved for families at the cost of precision—is, to our knowledge, the first quantified demonstration of this tension in dementia care LLM applications and is consistent with broader cautions about LLMs in health care communication [32,33].

### Limitations

Several limitations should be considered when interpreting these results.

Generalizability of the findings is constrained along 3 specific dimensions. First, the VR environment library comprises 5 scenes drawn from a specific production context: these environments sample a narrow slice of the space of possible dementia care VR content, and results should not be extrapolated to commercial or substantially larger libraries without separate evaluation. Second, the 10-dimension semantic encoding used to represent environments was derived inductively from this specific library; while the dimensions are plausible on a priori grounds, their mapping onto different content—therapeutic reminiscence scenes, interactive games, music-driven experiences—has not been validated, and the transferability of the encoding protocol itself remains an open question. Third, the study cohort comprises 21 residents in a single long-term care facility in Québec, predominantly with Alzheimer disease, at ISO-SMAF levels 7 to 10 (marked to severe functional disability); findings cannot be assumed to generalize to earlier disease stages, different cognitive etiologies, or different cultural and care system contexts. Replication across multiple sites, broader cohorts, and independent content libraries is a necessary prerequisite to any clinical recommendation.

Only 4 of 5 environments appeared in the test period (weeks 8‐10); the beach environment was not scheduled during those weeks. All ranking evaluations are therefore 4-way comparisons, and the model’s ability to recommend the missing environment remains untested.

The environment permutation test yielded a *P* value of .045 (*P*=.04 with 2000 permutations)—statistically significant but borderline, obtained from a single model configuration, and accompanied by a top-1 hit rate CI (19.8%‐35.7%) that includes the chance baseline. In addition, as stated in the Methods section, the choice of the focal configuration was partly informed by the test-set environment permutation result, so the test set is not fully independent for this inference. Shuffling feature columns after training cannot retroactively change what the model learned, so the test remains informative about whether the trained model uses environment information; what it cannot provide is an unbiased estimate of how often such a signal would be detected in a configuration chosen without reference to the test set. The remedy—a selection criterion defined on training period data only—was applied at revision: it does not select the compact model, and no retained configuration shows significant environment sensitivity on validation folds (Table 3). The test set result therefore stands as an exploratory finding, and the property that motivated the focal choice was not reproducible from training period data. We interpret the present results as preliminary evidence that an environment-level signal may exist—its direction was consistent across labelings and seeds—not that it is large, robust, or clinically useful.

The hybrid target is a constructed criterion rather than a validated well-being measure. Its within-person median split labels approximately half of each participant’s sessions as physiologically “better” by construction, irrespective of the magnitude of the underlying differences in RMSSD, and labels for sessions close to a participant’s median are sensitive to measurement noise. The sensitivity analyses (Table 5) show that the label itself is robust to the threshold (κ=0.79‐0.89) but that the environment permutation significance is not: alternative thresholds and a continuous target preserved the direction of the effect without reaching significance. Two further caveats apply: the threshold analysis rescored a model trained on the median-split labels rather than retraining per threshold, which may penalize the alternative labelings; and the within-person median was computed over all sessions, including the test period, so training labels depend slightly on test period data. “Calm” should therefore be read strictly as the operational label defined in the Methods section, and not as a direct measure of the resident’s subjective state.

Sense of presence was not measured. Between-environment differences in physiological response could therefore reflect differences in presence or immersion—driven, for example, by camera motion, visual complexity, or the interactive element of active forest—rather than differences in comfort per se. The semantic encoding captures some of these properties, but the present design cannot separate presence from calm. Questionnaire-based presence measures are impractical in this population, and neurophysiological presence indices [25,26] have so far been validated only in cognitively intact adults; adapting them to residents with major neurocognitive conditions would be a prerequisite for disentangling the 2 in future work.

Sensory status was characterized only by recorded use of corrective lenses and hearing aids. Hearing was not formally assessed, and because only one participant used a hearing aid in a population in which hearing loss is common, the audio dimensions of the environment encoding (nature sounds and music) may have been perceived unevenly across residents, which could contribute to their weak or negative permutation importance.

LOPO cross-validation showed high variability (MAE range 0.00‐1.00), indicating that some individuals’ profiles are not well predicted from the patterns of the remaining 20 residents. The test period (weeks 8‐10) may also be subject to temporal confounding from habituation effects, seasonal changes, or progressive cognitive decline.

Finally, all report evaluation was automated (LLM-as-judge). While this approach enables systematic evaluation at scale, it has not been validated against the judgments of actual care staff or family members. Human evaluation is planned as future work.

A residual ethical question, distinct from the procedural safeguards described in the Ethical Considerations section, concerns what consent can meaningfully mean for continuous physiological monitoring in a population that cannot fully articulate its preferences. Institutional approval and per-session willingness verification address the procedural dimension of this question but do not resolve the deeper concern that decisions about participation are necessarily made on behalf of residents rather than by them. This caveat extends to any future deployment of the recommendation pipeline beyond a research context.

Two deployment-level risks warrant attention before any clinical translation of this pipeline. The first is care-staff overreliance on algorithmic recommendations. A system that outputs a ranked list of “calmest environments” for each resident has the potential to displace rather than support staff judgment, particularly in time-pressured care settings; any prospective deployment will require safeguards that preserve staff discretion, including explicit communication of model uncertainty and clear override protocols. The second concerns the report-generation component. The fidelity-compliance trade-off documented in the RQ3 results—where reports passed all binary safety checks while drifting toward clinical register for staff audiences and sacrificing numerical precision for family audiences—illustrates that LLM-generated text can satisfy structured safety filters yet still misrepresent a resident’s experience in ways that are not immediately obvious to a nontechnical reader. Routine human review of generated reports, at least during initial deployment periods, should be a precondition for any operational use.

The economic case for this approach turns on a simple substitution: if matched environment selection reduces the frequency of distress episodes that currently trigger pharmacological response or one-on-one care, the wearable infrastructure and pipeline costs could be offset by reductions in those downstream interventions. This study does not test that substitution. A formal cost-effectiveness analysis alongside any prospective validation trial is identified as necessary future work.

### Conclusions

This feasibility and proof-of-concept study shows that a complete decision-support pipeline—from wearable physiological sensor to hybrid calm target to data-driven environment ranking to plain-language report—is technically implementable for VR comfort sessions in residential dementia care. An environment-level signal was detectable (permutation *P*=.045; *P*=.04 with 2000 permutations), person-specific, and interpretable, with water presence emerging as the most influential environment characteristic. However, the signal was small, borderline in statistical terms, and obtained from a single configuration whose selection was partly informed by the same test set; its significance did not survive alternative target definitions in sensitivity analyses, although its direction did. It should be regarded as preliminary evidence requiring independent replication, not as evidence of validated clinical usefulness. A compact model using 23 features outperformed larger models not in prediction accuracy but in its ability to use environment information—a finding that underscores the value of feature parsimony when the RQ concerns a small, specific signal.

The system is not intended to replace clinical judgment. Its purpose is to surface candidate patterns that are difficult to see unaided—for example, that one resident may relax more in environments with water while another may prefer social density—and to communicate them transparently so that staff can weigh them against their own observations.

Prospective validation is the necessary next step. A suitable study would enroll approximately 80 to 120 residents across 3 to 5 long-term care facilities to provide adequate power for multisite inference, with a stepped-wedge or cluster-randomized design (avoiding the practical complications of per-resident randomization in long-term care routines). The primary outcome should be an objective index of in-session distress—for example, PAS-2 vocal-agitation episode frequency per session, or a prespecified hybrid behavioral-physiological endpoint—with the comparator being usual care environment rotation or staff-selected scheduling. Ethical oversight should follow the substitute decision-maker framework applied here (see Limitations section), extended to a multisite consent protocol. Until such a study is completed, the present pipeline should be regarded as a demonstration of technical feasibility, not as a clinical tool ready for deployment.

Getting the data-to-report pipeline to work end-to-end, across 21 residents and 420 sessions, in a real long-term care setting, was the central contribution of this work; obtaining preliminary evidence that an environment signal may be recoverable from such data is a modest step toward dementia care systems that listen to the resident’s body, not just the observer’s notes—a step whose value depends on the independent replication we have identified as the necessary next test.

## Supplementary material

10.2196/101192 Multimedia Appendix 1 Methodological specifications: semantic encoding rubric, hypersearch grid, report quality evaluation rubric, and preprocessing or quality control criteria.

10.2196/101192 Multimedia Appendix 2 Study design and environment exposure, data quality and feature coverage, physiological signal processing, focal-model diagnostics (predicted vs observed values, longitudinal trajectories, and label-permutation null distribution), and report evaluation compliance summary.

## Abbreviations

BPSD: behavioral and psychological symptoms of dementia
EDA: electrodermal activity
EEG: electroencephalography
GSR: galvanic skin response
HR: heart rate
HRV: heart rate variability
ISO-SMAF: ISO-classified Système de mesure de l’autonomie fonctionnelle (functional autonomy measurement system)
LLM: large language model
LOPO: leave-one-participant-out
MAE: mean absolute error
PAS: Pittsburgh Agitation Scale
PAS-2: Pittsburgh Agitation Scale, second version
pNN50: percentage of successive RR intervals differing by more than 50 ms
PPG: photoplethysmography
QUALID: Quality of Life in Late-Stage Dementia
RCT: randomized controlled trial
REB: Research Ethics Board
RMSSD: root mean square of successive differences (between adjacent RR intervals)
RQ: research question
RSA: respiratory sinus arrhythmia
SDNN: SD of normal-to-normal RR intervals
VR: virtual reality
