# Supplementary material for: Data-Driven Virtual Reality Environment Recommendation for Long-Term Care Residents With Major Neurocognitive Conditions: Feasibility and Proof-of-Concept Study
Source: JMIR XR Spat Comput. 2026 Nov 12;3:e101192. doi: 10.2196/101192 (PMC13759178; doi:10.2196/101192)
Supplement: Supplementary file 1 [file xr-v3-e101192-s001.docx]

This appendix provides the full specifications for three methodological components referenced in the main manuscript: (A) the 10-dimension semantic encoding rubric used to characterize VR environments; (B) the hyperparameter search grid used during model selection; and (C) the nine-dimension rubric used for automated evaluation of generated reports.

## Section A: Semantic Environment Encoding Rubric

Each of the five VR environments was characterized by two independent raters along ten ordinal semantic dimensions, each rated from 0 to 3 with the anchors below [1]. Raw values were normalized to the unit interval by dividing by 3 prior to use as model input features. Inter-rater discrepancies were resolved through discussion-based consensus given the small number of items (5 environments × 10 dimensions = 50 cells); independent pre-reconciliation ratings were not retained in machine-readable form (acknowledged as a limitation in the main manuscript).

### A.1 Dimension definitions

**Camera motion.** Rate of change of the camera viewpoint relative to the virtual world, independent of within-scene object movement. 0 = stationary or near-stationary viewpoint; 1 = slow drift or occasional reorientation; 2 = sustained moderate motion (e.g., walking pace); 3 = rapid or continuous motion.

**Scene motion.** Movement of objects, characters, or natural elements within the scene independent of the camera. 0 = static scene; 1 = subtle motion (e.g., gentle foliage); 2 = moderate animation (e.g., flowing water, walking figures); 3 = pervasive dynamic motion.

**Audio: nature.** Prominence of natural ambient sound (wind, water, birds, foliage). 0 = absent; 1 = faint background; 2 = clearly audible and continuous; 3 = dominant component of the soundscape.

**Audio: music.** Prominence of musical content (instrumental or vocal music distinguishable as composed). 0 = absent; 1 = faint background; 2 = clearly audible and continuous; 3 = dominant component of the soundscape.

**Social presence.** Apparent presence of human or human-like figures in the scene (interactive or non-interactive). 0 = none visible; 1 = distant or peripheral figures; 2 = mid-distance figures plausibly co-present with the viewer; 3 = close, foregrounded social presence.

**Visual openness.** Perceived spatial expansiveness of the visible scene, including sky exposure, distant horizons, and absence of enclosing structures. 0 = enclosed (walls, ceiling, dense vegetation); 1 = partially open; 2 = open (e.g., field, plaza); 3 = expansive (e.g., open ocean, broad vista).

**Water presence.** Presence and prominence of water bodies in the scene. 0 = none; 1 = small or peripheral water (e.g., distant pond); 2 = moderate water presence (e.g., stream, fountain); 3 = dominant water feature (e.g., flowing river, lake, ocean).

**Interaction demand.** Extent to which the scene appears to invite or require viewer participation (interactive controls, gaze targets, decision points). 0 = passive viewing only; 1 = optional ambient interaction; 2 = recurrent prompts to act; 3 = continuous interactive demands.

**Visual complexity.** Density and heterogeneity of visual elements (count of distinct objects, textural variation, edge density). 0 = sparse, uniform scene; 1 = moderate detail; 2 = dense scene with substantial variation; 3 = highly complex, information-rich visual field.

**Altitude.** Apparent vertical position of the viewpoint relative to the ground plane. 0 = ground-level; 1 = elevated near ground (e.g., low hill, deck); 2 = mid-elevation viewpoint; 3 = high vantage point (e.g., aerial, mountaintop).

### A.2 Consensus values per environment (Table A1)

The table reproduces the consensus 0–3 ratings used in the main manuscript (see also Table 2, which presents the normalized values).

| Environment | Cam | Scn | Nat | Mus | Soc | Opn | Wat | Int | Cpx | Alt |
| --- | --- | --- | --- | --- | --- | --- | --- | --- | --- | --- |
| Beach | 1 | 1 | 1 | 0 | 1 | 2 | 2 | 0 | 1 | 1 |
| Île d’Orléans | 3 | 1 | 0 | 3 | 0 | 2 | 1 | 0 | 2 | 2 |
| Forest & stream | 3 | 2 | 2 | 0 | 1 | 1 | 3 | 0 | 3 | 1 |
| Old Québec | 2 | 2 | 1 | 0 | 3 | 1 | 0 | 0 | 2 | 1 |
| Active forest | 2 | 2 | 2 | 0 | 0 | 1 | 1 | 1 | 3 | 1 |

*Note.* Dimension abbreviations: Cam = camera motion; Scn = scene motion; Nat = audio (nature); Mus = audio (music); Soc = social presence; Opn = visual openness; Wat = water presence; Int = interaction demand; Cpx = visual complexity; Alt = altitude.

## Section B: Hypersearch Grid Specification

The full hyperparameter search evaluated 2,108 configurations across the seven dimensions described below. Selection was by mean cross-validation MAE (3-fold expanding-window cross-validation within weeks 1–7; training unseeded), with held-out test-set performance reported subsequently for the configurations retained for full evaluation.

### B.1 Sequence architectures (5 variants)

| ID | Architecture |
| --- | --- |
| S1_lstm | LSTM recurrent encoder |
| S2_gru | GRU recurrent encoder |
| S3_transformer_lite | Lightweight Transformer encoder |
| S4_ridge | Ridge regression baseline (sklearn) |
| S5_random_forest | Random forest baseline (sklearn) |

The S0_baseline label in some logs refers to a population-mean predictor used for sanity-checking; it is excluded from the configuration count.

### B.2 Prediction targets (7 variants)

| ID | Target |
| --- | --- |
| T1_vocal_only | PAS-2 vocal intensity (continuous) |
| T2_agitation_sum | Sum of PAS-2 agitation subscale items (continuous) |
| T3_binary_calm | Binary indicator of behavioral calm (PAS-2 vocal = 0) |
| T4_hybrid_rsa_lomb | Hybrid: behavioral calm AND elevated RSA (Lomb estimator) |
| T5_hybrid_rsa_welch | Hybrid: behavioral calm AND elevated RSA (Welch estimator) |
| T6_hybrid_rmssd | Hybrid: behavioral calm AND elevated RMSSD |
| T7_hybrid_pnn50 | Hybrid: behavioral calm AND elevated pNN50 |

For each hybrid target Tk, the corresponding source physiological feature was excluded from input features at training time to prevent target leakage.

### B.3 Feature sets (6 variants)

| ID | Description | Approximate count |
| --- | --- | --- |
| F1_all | Physiology + environment + profile + behavioral observations + text embeddings | 370 |
| F2_no_qualid | F1 minus QUALID behavioral items | 367 |
| F3_physio_only | Physiological features only (ablation baseline) | 98 |
| F4_no_physio | F1 minus physiological features | 272 |
| F6_env_only | 10 semantic environment features only | 10 |
| F7_reduced | Compact curated subset (10 physio + 3 behavioral + 10 env) | 23 |

The F5_no_environment variant was removed during Amendment 5; F3_physio_only serves as the ablation baseline reported in the main manuscript.

### B.4 Loss functions (4 variants)

| ID | Loss |
| --- | --- |
| L1_mse | Mean squared error |
| L2_ordinal | Ordinal regression loss (Frank-Hall encoding) |
| L3_crossentropy | Multi-class cross-entropy |
| L4_binary_crossentropy | Binary cross-entropy (used for T3 and hybrid targets) |

### B.5 History lengths (4 variants)

| ID | History |
| --- | --- |
| H1_all | All preceding sessions for the participant |
| H2_last5 | Most recent 5 sessions |
| H3_last3 | Most recent 3 sessions |
| H4_current_only | Current session only (no history) |

### B.6 Imputation strategies (2 variants)

| ID | Strategy |
| --- | --- |
| I1_median_plus_indicators | Per-feature median fill plus binary missingness indicator columns |
| I2_zero_binary_median_continuous | Zero-fill for binary features and median-fill for continuous features |

### B.7 Continuous hyperparameters

The following continuous hyperparameters were swept on a grid for Keras-based architectures (S1, S2, S3):

- Hidden dimension: {32, 64, 128, 256}
- Dropout rate: {0.0, 0.1, 0.2, 0.3}
- Learning rate: {1e-4, 5e-4, 1e-3, 5e-3}
- Weight decay: {0.0, 1e-4}
- Number of layers: {1, 2}

Sklearn baselines (S4, S5) used architecture-appropriate continuous parameters (regularization strength for ridge; tree count and depth for random forest) not enumerated here.

### B.8 Selection criterion and final focal model

Mean cross-validation MAE across the three expanding-window folds was the primary ranking criterion; the retained configurations were the best representative of each (target, feature set) pair among environment-aware feature sets, plus the best physiology-only configuration. The selected focal model (used for all results in the main manuscript unless otherwise stated):

| Setting | Value |
| --- | --- |
| Architecture | S3_transformer_lite |
| Target | T6_hybrid_rmssd |
| Feature set | F7_reduced (23 features) |
| Loss | L4_binary_crossentropy |
| History | H1_all |
| Imputation | I2_zero_binary_median_continuous |
| Hidden dimension | 64 |
| Dropout | 0.3 |
| Learning rate | 0.001 |
| Weight decay | 0.0 |
| Number of layers | 1 |

The corresponding ablation baseline reported in the main manuscript uses the same architecture family with feature_set = F3_physio_only (98 features; no environment or behavioral characteristics), hidden dimension 128 and dropout 0.1, as selected by the search.

## Section C: Report Quality Evaluation Rubric

Each generated report (60 reports across 30 sessions, two audience variants per session) was evaluated along nine dimensions. Two are binary compliance checks performed by deterministic rule-based evaluators; three are quantitative metrics computed against session ground-truth data; four are qualitative ratings produced by an automated grader (a separate large language model serving as judge). All quantitative and qualitative dimensions were computed on a per-report basis and aggregated across audience type for the analyses in the main manuscript.

### C.1 Binary compliance checks (2 dimensions)

**Medical compliance (binary).** Pass if the report contains no diagnostic or clinical-assessment language (e.g., terms such as “diagnosis”, “condition”, “disease”, “treatment”, “symptoms”, or first-person clinical attributions). Implemented as a curated keyword check against a domain-specific exclusion list.

**Information sufficiency (binary).** Pass if the report contains all three required structural components: (i) a session summary, (ii) an environment recommendation for the next session, and (iii) at least one cited evidence item supporting the recommendation. Implemented as a structural parser against the prompt-defined report template.

### C.2 Quantitative metrics (3 dimensions)

**Numerical fidelity rate.** For each numerical claim in the report (e.g., “RMSSD increased by 12% relative to last session”), the claimed value is compared against the corresponding ground-truth value extracted from the session data. The rate is the proportion of verifiable claims whose reported value matches ground truth within a tolerance of ±5% relative difference (continuous quantities) or exact match (categorical quantities).

**Evidence coverage rate.** For each substantive claim that requires support (recommendations, comparative statements, attributions), the rate is the proportion that include at least one cited evidence item from the session evidence trace.

**Citation density.** Count of distinct evidence citations divided by the count of substantive claims in the report. A density of 1.0 indicates one citation per claim on average.

### C.3 Qualitative Likert ratings by automated grader (5 dimensions)

A separate large language model served as the automated grader, prompted with a chain-of-thought form-filling rubric requiring the grader to produce both a numerical score (1–5) and a brief written justification per dimension. The five qualitative Likert dimensions are defined as follows.

**Clarity (1–5).** The extent to which the report is intelligible to its intended audience (family or staff) without prior context. 1 = unclear or jargon-heavy; 3 = adequate; 5 = consistently transparent.

**Usefulness (1–5).** The likely utility of the report for its intended downstream use: caregiver awareness for family reports; documentation and planning support for staff reports. 1 = of little practical use; 3 = reasonably useful; 5 = directly serviceable for the audience’s purpose.

**Actionability (1–5).** The extent to which the report’s recommendations are concrete and implementable by the intended audience without further interpretation. 1 = vague or non-actionable; 3 = partially actionable; 5 = directly actionable.

**Fidelity (1–5).** A grader-rated qualitative judgment of how faithfully the prose reflects the underlying session data, complementing (and partly redundant with) the deterministic numerical-fidelity rate in Section C.2. 1 = substantive deviations from source data; 3 = generally faithful with minor inaccuracies; 5 = consistently faithful throughout.

**Non-medical compliance (1–5).** The degree to which the report’s register and framing avoid clinical, diagnostic, or prescriptive language at a register level beyond the binary forbidden-terms filter. 1 = clinical register throughout; 3 = mixed register; 5 = consistently non-medical.

### C.4 Use and interpretation in the main manuscript

The qualitative ratings produced by an LLM-as-judge are subject to documented biases (position effects, verbosity preference, self-enhancement) that motivate conservative interpretation [2,3]. As discussed in the RQ3 results in the main manuscript, these ratings are presented as exploratory feasibility evidence rather than as definitive quality measurements; substantive conclusions about report quality are drawn primarily from the binary compliance checks and the quantitative metrics, with qualitative ratings serving as corroborating descriptive context.

## Section D: Preprocessing Parameters and Quality-Control Criteria

This section summarizes the per-session signal preprocessing and quality-control pipeline applied to physiological recordings before feature extraction. The pipeline operates separately on PPG (cardiac) and EDA (electrodermal) channels, retains a per-window usability flag for downstream auditing, and produces session-level summary features only from windows that passed the channel-specific QC criteria.

### D.1 PPG channel (cardiac signals)

Raw photoplethysmography signals from the wrist-worn sensor were first interpolated onto a uniform time grid and bandpass-filtered to retain the cardiac frequency band. Beat detection was performed on the filtered signal using an adaptive peak-finding procedure with refractory constraints derived from physiologically plausible heart rate bounds (40–180 bpm). Inter-beat intervals (IBI) were extracted from successive peak times and screened for ectopic and artefactual beats using a relative-difference threshold against the surrounding local median.

Quality control was performed on overlapping fixed-duration analysis windows. Each window was flagged as usable only if all of the following held: (i) sufficient density of accepted beats within the window; (ii) the within-window heart rate from the wearable’s onboard estimator agreed with the beat-derived heart rate to within a stipulated tolerance; (iii) accelerometer-derived motion magnitude during the window did not exceed the motion-artifact threshold; and (iv) no flatline or saturation segments were detected. Windows that failed any criterion were excluded from feature computation but retained as audit variables (session-level usable-window percentage, motion-artifact fraction, and device–algorithm HR disagreement) and preserved as metadata at the session level.

For each usable window, the following features were extracted: mean and standard deviation of heart rate; mean, standard deviation, and RMSSD of inter-beat intervals; SDNN; pNN50; and respiratory sinus arrhythmia (RSA) high-frequency power computed using both Welch periodogram and Lomb–Scargle methods on resampled IBI series. Per-window features were aggregated to the session level by taking the mean (and, where applicable, the standard deviation) across usable windows.

### D.2 EDA channel (electrodermal activity)

Raw EDA signals were interpolated onto a uniform time grid and decomposed into tonic (slow) and phasic (fast) components using a low-pass / high-pass split with channel-appropriate cutoff frequencies. Window-level QC for EDA applied additional checks specific to skin-conductance signals: flatline detection (within-window standard deviation below a stipulated threshold), spike rejection (z-score outlier detection against the local distribution), and slope-rate sanity bounds against physiologically plausible response dynamics. Negative EDA epsilon constraints were applied to handle edge cases at session start.

Per-window EDA features included tonic mean and standard deviation, phasic RMS, and skin-conductance response (SCR) count using a peak-detection routine with minimum-peak-distance and amplitude criteria appropriate to the sampling rate. Aggregation to the session level mirrored the PPG procedure.

### D.3 Session inclusion criteria

A session was included in subsequent modeling if at least one channel produced any usable windows. PPG passed quality thresholds in 371 of 374 recorded sessions (99.2%). EDA had a lower pass rate (54%, as reported in the original feature-coverage diagnostics) due to the higher prevalence of motion artefacts and electrode-contact issues with the wrist-worn EDA sensor in this population. The hybrid calm target (used in the focal model) requires only that an RMSSD-bearing PPG session is present and that a PAS-2 vocal intensity rating is available; consequently the EDA pass rate is not a limiting factor on the per-session inclusion criterion for the focal target.

### D.4 Reproducibility note

The full set of QC parameter values (window lengths, step sizes, cutoff frequencies, motion thresholds, beat-detection refractory constraints, and rejection criteria) was held constant across all participants and sessions and is encoded in the analysis pipeline configuration files used for this study. Specific numeric parameter values are available from the corresponding author upon reasonable request.

## References

1. Neveu E, Huynh LK, Roy I, et al. Social cognition assessment using virtual reality: A systematic review. Technol Mind Behav. 2025;6(3). doi:10.1037/tmb0000159

2. Zheng L, Chiang WL, Sheng Y, et al. Judging LLM-as-a-judge with MT-Bench and Chatbot Arena. Adv Neural Inf Process Syst. 2023;36. https://arxiv.org/abs/2306.05685

3. Liu Y, Iter D, Xu Y, Wang S, Xu R, Zhu C. G-Eval: NLG evaluation using GPT-4 with better human alignment. Proc Conf Empir Methods Nat Lang Process. 2023:2511–2522. doi:10.18653/v1/2023.emnlp-main.153
