# Supplementary material for: Data-Driven Virtual Reality Environment Recommendation for Long-Term Care Residents With Major Neurocognitive Conditions: Feasibility and Proof-of-Concept Study
Source: JMIR XR Spat Comput. 2026 Nov 12;3:e101192. doi: 10.2196/101192 (PMC13759178; doi:10.2196/101192)
Supplement: Supplementary file 2 [file xr-v3-e101192-s002.docx]

This appendix contains additional diagnostic and exploratory figures generated during the analysis. None are essential for understanding the main results, but each provides useful supporting context for readers interested in study design (Figures S1–S2), data quality (Figures S3, S5–S7), signal processing detail (Figure S4), model behavior (Figures S8–S10), or report-evaluation diagnostics (Figure S11). Internal figure titles within the images reflect the original analysis-pipeline labeling and may differ from the figure numbers used in this appendix; the figure number used in this manuscript is given in each caption.

### Figure S1 — Problem framing diagram

Figure S1

**Figure S1.** Schematic of the data-driven VR environment recommendation problem addressed in this study. A resident with a given dementia profile and physiological response history is presented with one of five immersive VR environments; the resulting behavioral response is observed via PAS-2 ratings. The decision-support agent uses the resident’s accumulated history of (environment, response) pairs to predict which environment is most likely to produce calm in the next session and ranks the five candidate environments accordingly.

### Figure S2 — Study timeline: environment assignment per week

Figure S2

**Figure S2.** Per-participant environment exposure across the 10-week study period. Each row is a participant; each column a week; cell color encodes which of the five VR environments was scheduled. Beach was not scheduled during weeks 8–10 (the test period for the focal model), as discussed in the Methods and Results. The figure visualizes both the rotation schedule and the per-participant variation in the realized exposure pattern.

### Figure S3 — Feature coverage by family

Figure S3

**Figure S3.** Proportion of sessions for which each feature family was successfully computed (passed quality control). Profile features (demographics, ISO-SMAF) have the highest coverage at 98%; PPG-derived features (HR, IBI/HRV, RSA) were obtained for 88% of sessions (371/420), PAS-2 ratings for 85% (358/420), and QUALID ratings for 95% (400/420); EDA has the lowest coverage at 52% (218/420), reflecting motion artefacts and electrode-contact issues with the wrist-worn EDA sensor in this population. Coverage rates were computed across all 420 scheduled sessions; thresholds and per-channel QC criteria are detailed in Multimedia Appendix 1, Section D.

### Figure S4 — PPG processing pipeline schematic

Figure S4

**Figure S4.** Block diagram of the photoplethysmography processing pipeline used to extract cardiac features. Raw PPG signals from the wrist-worn sensor are interpolated, bandpass-filtered, and passed through adaptive peak detection to extract beats; inter-beat intervals are screened for artefacts; per-window features (HRV time- and frequency-domain measures, RSA) are computed on usable windows only; and session-level summary statistics aggregate across windows. Full parameter values for each block are summarized in Multimedia Appendix 1, Section D.

### Figure S5 — Example annotated session trace

Figure S5

**Figure S5.** Illustrative single-session trace for one participant, showing the synchronized physiological time series (PPG-derived heart rate, EDA tonic and phasic components) alongside session-level metadata. The figure illustrates the within-session signal characteristics that inform the per-window QC and feature-extraction procedures described in Multimedia Appendix 1, Section D. Specific identifiers are anonymized.

### Figure S6 — Per-participant session coverage heatmap

Figure S6

**Figure S6.** Per-participant session count by week, with cell shading indicating successful PPG coverage. The figure complements Figure S3 by showing that PPG coverage is reasonably uniform across participants (no participant is systematically excluded) but heterogeneous across weeks, consistent with the operational realities of physiological monitoring in long-term care.

### Figure S7 — Feature distributions across the cohort

Figure S7

**Figure S7.** Distribution of the principal physiological features across all sessions in the analytic dataset (HR mean, RMSSD, SDNN, EDA tonic mean, EDA phasic RMS). Distributions are right-skewed for HRV measures and approximately log-normal for EDA tonic levels, as expected for these signals in older adult populations. The figure provides reader-assessable context for the normalization and feature-engineering choices described in the Methods.

### Figure S8 — Predicted vs. observed scores on the test set

Figure S8

**Figure S8.** Predicted hybrid-target scores from the focal 23-feature model plotted against observed values on the 108 labeled held-out test sessions (weeks 8–10). The figure illustrates both the calibration of the model’s predictions and the residual structure: predictions are well-calibrated overall but exhibit greater variance at intermediate hybrid-target values, consistent with the difficulty of differentiating mid-range states using behavioral observation alone.

### Figure S9 — Case timeline plots

Figure S9

**Figure S9.** Per-participant longitudinal trajectories for three representative residents, showing observed PAS-2 vocal intensity (red circles) and the focal model’s predicted score for the resident’s actual environment at each session (blue squares) across all weeks. Vertical band shading encodes the actual environment scheduled at each session. The figure provides qualitative reader-assessable evidence of how the model’s per-session predictions track behavioral observations across the 10-week study.

### Figure S10 — Label-permutation null distribution

Figure S10

**Figure S10.** Null distribution of the focal model’s test MAE under random permutation of the hybrid-target labels (30 permutations, each with a fresh model retrained on the shuffled labels). The observed test MAE on the un-permuted labels (0.247) lies within the null distribution (P=.45): the model’s aggregate predictive accuracy does not exceed what can be achieved by fitting on shuffled targets. Evidence for the environment-level signal rests on the environment permutation test reported under RQ1, not on this control; the interpretation of the two tests together is discussed in the Results.

### Figure S11 — Report-evaluation compliance summary

Figure S11

**Figure S11.** Pass/fail summary of the binary compliance checks across the 60 generated reports (30 sessions × 2 audience variants). Both checks (medical-language compliance and length compliance) pass at 100%, supporting the descriptive feasibility claim made in the RQ3 results. The five Likert-scale ratings produced by the LLM judge are reported in Table 6 of the main manuscript.

# References

This appendix contains supplementary figures only; no external sources are cited. All references for analytical procedures, instruments, and prior work informing the figures presented here are listed in the reference list of the main manuscript.
